# Correlating Structural Disorder and Pr^3+^ Emission Dynamics in Lu_3_Al_2.5–*x*_Sc_*x*_Ga_2.5_O_12_ Crystals: A Comprehensive Structure–Property Investigation

**DOI:** 10.1021/acsomega.5c01062

**Published:** 2025-05-08

**Authors:** Karol Bartosiewicz, Wioletta Dewo, Vitali Nagirnyi, Tomasz Runka, Marco Kirm, Takahiko Horiai, Damian Szymanski, Akihiro Yamaji, Shunsuke Kurosawa, Paweł Socha, Jan Pejchal, Vladimir Babin, Robert Kral, Aleksei Kotlov, Akira Yoshikawa, Martin Nikl

**Affiliations:** † Institute of Physics, Academy of Sciences of the Czech Republic, Na Slovance 1999/2, Praha 18200, Czechia; ‡ Faculty of Materials Engineering and Technical Physics, 49632 Poznan University of Technology, Piotrowo 3, Poznań 60965, Poland; § Institute of Physics, 37546 University of Tartu, W. Ostwald Str. 1, Tartu 50411, Estonia; ∥ New Industry Creation Hatchery Center, 13101 Tohoku University, 2-1-1 Katahira Aoba-ku, Sendai, Miyagi 9808577, Japan; ⊥ Institute of Low Temperature and Structure Research, Polish Academy of Sciences, Okolna 2, Wrocław 50422, Poland; # Institute for Materials Research, Tohoku University, 2-1-1 Katahira Aoba-ku, Sendai, Sendai, Miyagi 9808577, Japan; ¶ Institute of Laser Engineering, Osaka University, 2-6 Yamadaoka, Suita, Osaka 5650871, Japan; ∇ Łukasiewicz Research Network—Institute of Microelectronics and Photonics, Aleja Lotników 32/46, Warsaw 02-668, Poland; ○ 28332 Deutsches Elektronen-Synchrotron DESY, Notkestr. 85, Hamburg 22607, Germany

## Abstract

This study explored the influence of Sc^3+^ ions incorporation on the structural, vibrational, luminescent, and scintillation properties of Pr^3+^-doped Lu_3_(Al, Sc)_2.5_Ga_2.5_O_12_ garnet crystals. Addressing the limited research on Sc-admixed and Pr^3+^ doped garnet systems, this work successfully demonstrated the crystallization of garnet crystals from the melt, overcoming the substantial atomic mismatch between Sc and Al while preserving the thermodynamic stability of the garnet phase. Importantly, Sc-admixing enhanced atomic homogeneity and allowed for increased doping concentrations of Pr^3+^ ions, which is crucial for tailoring the functional properties of advanced optical materials. The trap depths ranged from 1.63 eV (deep traps) to 0.22 eV (shallow traps) across all samples, with frequency factors predominantly between 1 × 10^7^ and 1 × 10^11^ s^–1^, consistent with first-order thermoluminescent kinetics. From a materials design perspective, Sc^3+^ ions substitution induced beneficial host lattice disorder, enhancing the emission intensity of 4f^1^5d_1_^1^ → 4f^2^ interconfigurational and 4f^2^ → 4f^2^ intraconfigurational transitions. This effect highlighted the potential of Sc as a promising substituent for enhancing the luminescence intensity of rare earth elements. Synchrotron radiation experiments provided insights into the impact of Sc on band gap energy and energy transfer efficiency toward Pr^3+^ ions offering new opportunities for engineering scintillators and phosphors with tunable optical properties.

## Graphical abstract

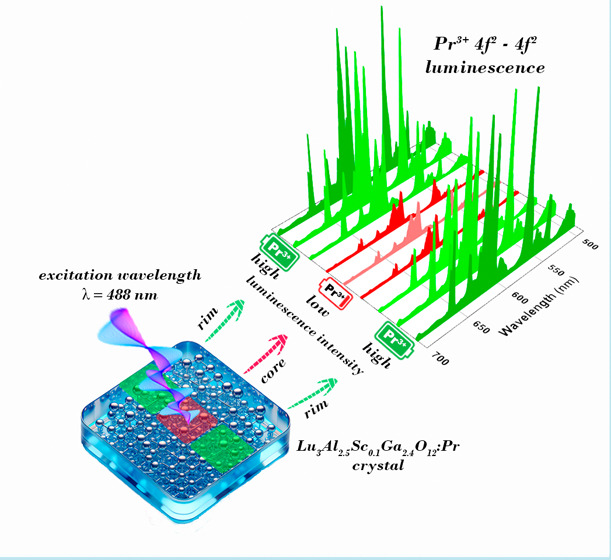

## 1 Introduction

The micropulling-down (μ-PD) method is widely used for the crystallization of numerous materials, including oxides, fluorides, semiconductors, and metals.^1,2^ The advantage of the method is its ability to have a high pulling rate, which allows the growth of more than one crystal per day, thus remarkably outperforming conventional growth techniques. The elemental distribution in crystals grown by μ-PD is controlled by several factors: (i) thermoelectromotive transfer of charged ion in the melt, (ii) the segregation coefficient, (iii) the geometry of the crucible, (iv) the liquid–solid interface, and (v) the composition of the melt.^1–4^ High flexibility in the modification of these factors in the μ-PD method has contributed to the development of multifunctional materials based on the garnet family.^2–8^ For instance, Lu_3_Al_5_O_12_ (LuAG) has attracted considerable attention as a host crystal for near-infrared solid-state lasers, as well as for optoelectronic devices, including computer memories and microwave optical elements, in medical surgery and imaging.^9–11^ Moreover, Lu_3_Al_5_O_12_ crystals doped with Ce^3+^ or Pr^3+^ ions can also serve as efficient scintillators in many applications due to their fast and bright emission originating from 4f^*N*–1^5d_1_^1^ → 4f^*N*^ interconfigurational transitions.^8^

The ideal crystal structure of Lu_3_Al_5_O_12_ is constituted by an uninterrupted, homogeneous, and highly organized sequence of Lu_3_-dodecahedra (LuO_8_), Al_2_-octahedra (AlO_6_), and Al_3_-tetrahedra (AlO_4_).^12^ The driving forces for the solubility or segregation of substituents are not solely determined by surface energy and configurational entropy; they are also influenced by the substituent’s size compatibility with the dodecahedral, octahedral, and tetrahedral sites of the garnet structure.^4,13–15^ This is evidenced in Lu_3_Al_5_O_12_ with a disordered crystal structure, wherein defect formation and an intricate substituent distribution across all polyhedra are observed upon doping.^8,12,13^ The relatively small ionic radius of Lu^3+^ (Lu^3+^_VIII_ = 0.977 Å) at the dodecahedral site^16^ strongly affects the solubility and segregation of dopants as well as defect formation processes during crystal growth.^8,12,13^ The defect formation is facilitated by the relatively high temperature (∼2200 K) of Lu_3_Al_5_O_12_ crystallization, which allows the Lu^3+^ (Lu^3+^_VI_ = 0.861 Å) ions to enter smaller crystallographic sites of the Al^3+^ (Al^3+^_VI_ = 0.535 Å) ions in the octahedral coordination.^13,17^ The Lu^3+^ ion at the octahedral Al^3+^ site creates shallow Lu^*x*^_Al_ antisite defects (AD). These trapping centers efficiently capture electrons from the conduction band, resulting in the formation of bound exciton states. Radiative recombination of such excitons produces luminescence in the ultraviolet spectral range. This process reduces the efficiency of energy transfer from the host lattice to Pr^3+^- and Ce^3+^-doping ions, resulting in a marked deterioration of the scintillation properties in garnet scintillators.^18^

During the crystallization process, incompatible substituents, introduced from the melt via the crucible orifice to the liquid–solid interface, exhibit limited retrograde diffusion into the melt.^2,4^ These substituents, predominantly excluded from incorporation at the crystallographic sites, undergo segregation toward the periphery of the molten zone. Subsequently, they are assimilated into the crystal structure, culminating in the formation of secondary phases.^14,19^ This results in the stabilization of the main crystal structure and radial composition gradients, while the axial elemental distribution becomes more homogeneous.^2,14,19^ Previous reports^13–15^ revealed that the ability to accumulate substituents in dodecahedral coordination depends mainly on the size of the cations forming the octahedral and tetrahedral sites of the garnet structure. Specifically, crystal lattices with larger atoms in the octahedral and tetrahedral sites can adopt a higher concentration of incompatible substituents in the dodecahedral sites. In contrast, increasing the concentration of larger incompatible atoms (compared to the native atoms) at the dodecahedral site significantly deteriorates the desired phase stability and reduces the elemental homogeneity of the single crystal.^14,15,19–22^

Uniform single crystals are important for many high-precision applications. Their production requires a good understanding of material congruent melting when the growing crystal has the same composition as the melt with a uniform distribution of atoms. The lattice constant was found to increase from 11.914 to 12.2371 Å in Lu_3_Al_5_O_12_ ^23^ and Lu_3_Ga_5_O_12_ ^24^ garnets, respectively, confirming the role of cationic radius in mediating complex garnet lattice expansion. Therefore, the expansion of the [AlO_6_] octahedra and [AlO_4_] tetrahedra in the structure of the Lu_3_Al_2_Al_3_O_12_ crystal could significantly improve the homogeneity of the crystal and radial distribution of the activator and therefore the overall functionality of the material. Substituting Al atoms in the [AlO_6_] octahedra (Al^3+^_VI_ = 0.535 Å) and [AlO_4_] tetrahedra (Al^3+^_IV_ = 0.39 Å) by larger Ga and Sc atoms leads to enlargement of the lattice constants and volume of the unit cell. In the Lu_3_Al_5_O_12_ host lattice, large Sc atoms are capable of occupying dodecahedral and octahedral coordination sites and form the [ScO_8_] (Sc^3+^_VIII_ = 0.87 Å) and [ScO_6_] (Sc^3+^_VI_ = 0.745 Å) structural units, respectively.^12,13,25,26^ Ga atoms preferentially occupy octahedral and tetrahedral coordination sites forming [GaO_6_] (Ga^3+^_VI_ = 0.62 Å) and [GaO_4_] (Ga^3+^_IV_ = 0.47 Å), structural units, respectively.^27,28^ The expansion of crystallographic lattice parameters facilitates an enhancement in the distribution coefficients of large-ionic-radius lanthanide species, notably Pr^3+^ and Ce^3+^ ions, thereby augmenting their incorporation efficacy within the host matrix. Another consequence of admixing large Ga and Sc elements, particularly relevant to scintillators, is a possible suppression of nonequivalent substitution of host atoms in octahedral sites, which may be responsible for the creation of defects acting as charge carrier traps. Furthermore, the associated changes in the crystal field lead to changes in energy levels of activator ions within the band gap and to changes in trapping states, which can noticeably modify the performance of the scintillation.^26,28^ Consequently, this interaction leads to the formation of Sc-bound excitons. Their radiative recombination causes bright UV emission centered around 275 nm, which overlaps with the 4f^2^ → 5d_1_^1^4f^1^ absorption bands of Pr^3+^ ions spanning between 260 and 290 nm.^26^ This spectral overlap facilitates an effective energy transfer pathway from the Sc-bound excitons to the Pr^3+^ ions, culminating in the amplification of the luminescence intensity and the enhancement of the overall scintillation efficiency of the material. Investigations on Sc^3+^, Pr^3+^-codoped Lu_3_Al_5_O_12_ (LuAG) epitaxial thin films have elucidated the significant influence of Sc^3+^ sensitizer concentration on the luminescence and scintillation properties of Pr^3+^ ions.^29,30^ By incrementally increasing the Sc^3+^ ion concentration up to 3 at. %, a concomitant enhancement in the emission intensity originating from the relaxation of the excited state of Pr^3+^ ions was observed, attributable to the efficient excitation energy transfer from Sc^3+^ ions. However, beyond the optimal 3 at. % Sc^3+^ concentration, a diminution in the emission intensity was noted. This phenomenon was tentatively ascribed to the formation of stable electron traps induced by the presence of excessive Sc^3+^ ions, which impeded the energy transfer processes.

Optical properties of rare earth (RE) ions located in the crystal lattice of garnets depend on electron–phonon interaction. Consequently, it is essential to ascertain the vibrational features of the host in which this ion resides.^1–8^ Considering the wide usage of garnet materials and the lack of information from vibrational spectroscopy of Pr^3+^-doped Lu_3_Al_2.5–*x*_Sc_*x*_Ga_2.5_O_12_ garnets, in particular, it is desirable to apply the Raman spectroscopy method to study their phonon spectra. Interestingly, the experimental data on the Sc impact on luminescence, scintillation, and structure properties of aluminum garnets have been limited up to now. Single crystals of (Gd,Y)_3_(Ga,Sc)_2_Ga_3_O_12_ doped with RE atoms have mainly been studied for laser, white LED, and scintillation applications.^5,25,29–33^ A review of the published literature has shown that the research devoted to the crystallographic, microstructure, Raman, luminescence, and scintillation properties of Pr^3+^ ions in single crystals of Sc-containing aluminum garnets is restricted to only a few reports.^5,12,32^

The primary objective of this investigation is to elucidate the systematic modulation of band gap energy in Lu_3_(Al,Ga)_5_O_12_ crystals through progressive Sc incorporation, building upon previously established Ga-induced modifications of the conduction band minimum (CBM).^27,34^ This research specifically examines the effect of Sc substitution on the further band gap engineering. The comprehensive study investigates the impact of systematic Sc incorporation in Pr^3+^-doped Lu_3_Al_2.5–*x*_Sc_*x*_Ga_2.5_O_12_ garnets across multiple critical parameters: (i) photoluminescence (PL) and scintillation characteristics of Pr^3+^ emission centers, (ii) vibrational spectroscopic features, (iii) perturbations in the host lattice configuration, (iv) crystal growth dynamics, and (v) radial compositional homogeneity of the synthesized crystals. The systematic variation of Sc^3+^ ion concentration enables detailed analysis of energy transfer mechanisms to Pr^3+^ ions and their subsequent impact on the luminescence and scintillation properties. A series of Pr^3+^-doped Lu_3_Al_5–*x*_Sc_*x*_Ga_2.5_O_12_ (Pr 0.1 at. %) single crystals with varying Sc^3+^ concentrations (*x* = 0.00, 0.10, 0.25, 0.50, 0.75, 1.00) were synthesized via the micropulling-down (μ-PD method).^1,35,36^ Structural characterization was conducted using powder X-ray diffraction (PXRD), while the crystal microstructure, elemental distribution, and Sc-induced lattice distortions were investigated through complementary SEM–EDS analysis and Raman spectroscopy. Optical absorption, PL, and scintillation parameters were evaluated as functions of the increasing Sc concentration. Thermoluminescence investigations were employed to characterize the Sc influence of the charged trap density and their influence on scintillation dynamics. This investigation discovers the fundamental role of controlled lattice disorder in modulating material properties, providing crucial insights for future materials design. The systematic approach to lattice disorder engineering presents novel opportunities for enhancing the material performance in applications spanning optoelectronics, solid-state illumination, and biomedical imaging.

## 2 Methodology

### 2.1 Crystal Growth Process

A series of Pr^3+^-doped Lu_3_Al_2.5–*x*_Sc_*x*_Ga_2.5_O_12_ single crystals where *x* = 0.00, 0.10, 0.25, 0.50, 0.75, 1.00 were grown from the melt using the μ-PD method^1,20^ with radiofrequency inductive heating. Across this series of crystals, the nominal concentration of Pr^3+^ ions was maintained at a constant value of 0.1 at. %. The starting materials were prepared by mixing 99.99% purity oxides of Lu_2_O_3_, Al_2_O_3_, Ga_2_O_3_, Sc_2_O_3_, and Pr_6_O_11_ from the Iwatani Corporation (Japan). The powder mixture was melted by inductive heating in an Ir crucible with a die of 3 mm diameter in a flowing Ar + O_2_ 2% atmosphere. Crystal growth was initiated in a LuAG seed oriented in the ⟨100⟩ direction. The pulling speed was 0.05 mm/min. The 1% weight surplus of Ga_2_O_3_ oxide was added to the starting materials to compensate for the evaporation of the Ga element. The Ga_2_O_3_ oxide excess was adjusted to the crystal growth conditions to provide an optimal crystallization process.

### 2.2 PXRD, SEM–EDS Analysis, and Raman Spectroscopy

Pieces of the grown crystals were crushed and ground into a powder in a mortar. PXRD analysis was performed in the 2θ range of 15–60° using a D8 DISCOVER-HS (BRUKER) diffractometer. Diffraction was measured using the Cu Kα radiation with a wavelength of approximately λ = 1.54 Å and a photon energy of *E* = 8.05 keV. The lattice constants and volume of the unit cells were estimated from the PXRD patterns using the approach described by Strocka et al.^37^ The cross-sectional morphology and chemical composition of Pr^3+^-doped Lu_3_Al_2.5–*x*_Sc_*x*_Ga_2.5_O_12_ single crystals with varying Sc^3+^ ion concentrations (for *x* ranging from 0.0 to 1.0) were analyzed using the NovaNanoSEM 230 scanning electron microscope (SEM) equipped with an energy-dispersive X-ray spectrometer Genesis XM4. SEM measurements were performed on gold-coated crystals mounted on carbon stubs. Coated samples were placed in the microscope chamber and analyzed using secondary electron (SE) and dispersive X-ray (EDS) signals to obtain high-quality SEM images (at an acceleration voltage of 3.0 kV) and EDS maps (at 30.0 kV), respectively. Due to limitations in detection sensitivity, quantitative analysis of the Pr element for all examined crystals and Sc elements for the Lu_3_Al_2.5–*x*_Sc_*x*_Ga_2.5_O_12_ crystal where *x* = 0.1 was not feasible using this technique. Consequently, EDS maps and line profiles for Pr and Sc elements for the abovementioned samples were not generated and are not discussed in this paper.

The nonpolarized Raman spectra (RM) were recorded at 300 K using a Renishaw inVia Raman microscope equipped with a thermoelectrically cooled CCD detector and an Ar^+^ laser operating at a wavelength of 514.5 nm. Raman spectra were recorded in the 100–900 cm^–1^ spectral range with a spectral resolution better than 2 cm^–1^. The laser beam with a power below 5 mW was focused on the sample with a ×50 objective. Peak positions were calibrated before data collection with a Si crystalline sample used as an internal standard. Spectral band parameters such as peak center position, intensity, integral intensity, and fwhm (half-maximum width) were determined after baseline correction using Wire 3.1 software fitting procedure. High spatial resolution luminescence spectra were also recorded with a Renishaw inVia Raman microscope. Two laser lines, i.e. 488 and 514.5 nm, as well as lenses with a magnification of ×50 were applied. The power of the laser beam was 0.1 and 2 mW, respectively.

### 2.3 Optical, Luminescence, and Scintillation Characteristics

The absorption spectra were measured with a JASCO V-730 instrument in the 200–800 nm spectral range at 300 K. Photoluminescence excitation (PLE) and emission (PL) spectra were studied under synchrotron radiation excitation in the vacuum ultraviolet spectral range. The emission spectra were not recorded for the *x* = 0.00 sample because it accidently detached from the sample holder during the measurements in the cryostat in the UHV chamber. The measurements were conducted using a luminescence setup at the P66 beamline of the PETRA III storage ring of DESY Photon Science (Hamburg, Germany).^38^ The P66 beamline has a focal spot ca. 3 × 0.5 mm on the sample, resulting in luminescence spectra without spatial resolution. The luminescence was recorded by a Kymera-328i UV–visible spectrometer with a grating (300 L/mm blazed at 300 nm), which is equipped with a Hamamatsu R6358 PMT and an ANDOR Newton 920 CCD camera mounted at different exit ports. The excitation spectra at 300 K were recorded in the 280–150 nm excitation range using an Al-coated grating (1200 L/mm blazed at 180 nm, a spectral resolution set to 0.65 nm) and were corrected to the incident photon flux by using the sodium salicylate signal. The typical spectral resolutions of the Kymera-328i device were 1 and 10 nm in emission and excitation spectral measurements, respectively. Radioluminescence spectra were measured under X-ray excitation (40 kV, 40 mA) (RINT2000, Rigaku). For the scintillation light yield (LY) and decay time measurements, the crystals were wrapped into several layers of Teflon tape and optically coupled to the light entrance window of an R7600 (Hamamatsu) photomultiplier tube (PMT) with optical grease. The high voltage was supplied by an ORTEC 556 unit, and the signals were read from the PMT anode. The signals passed a shaping time of 2 μs and were converted to digital signals by a Pocket MCA 8000A multichannel analyzer provided by Amptek Co. The scintillation decay curves were obtained using a digital oscilloscope Tektronix TDS3034B under excitation by 662 keV photons from the ^137^Cs radioisotope.

### 2.4 Thermoluminescence

Thermally stimulated luminescence glow curves were measured with a HORIBA Jobin-Yvon 5000 M spectrometer equipped with a liquid nitrogen cryostat (Oxford Instruments) and a TBX-04 (Hamamatsu) photomultiplier operating in the 200 and 800 nm spectral range. The samples were irradiated by an X-ray tube (Seifert) with a tungsten target operated at 40 kV. The dose obtained by the sample during irradiation is estimated to be about 450 Gy. The TL was recorded during linear heating of the sample with the 0.1 K/s rate in the range of 77,500 K.

## 3 Experimental Results and Discussion

### 3.1 Crystal Phase and Morphology

The examples of as-grown rods along with polished radial plates of Pr^3+^-doped Lu_3_Al_2.5–*x*_Sc_*x*_Ga_2.5_O_12_ crystals are shown in Figure 1a. The surface of a crystal without scandium is slightly opaque due to thermal etching and the formation of surface defects.^19,39^ The surface opacity vanished in the crystals containing Sc atoms. Some parts of the as-grown crystal rods were cracked. However, the cracks appeared mostly on the crystal surface, while the crystal core remained crack-free. To reduce the possibility of concentration differences caused by dopant segregation during growth, radial plates were cut at the same distance from the seed side of each grown rod. Only the rim of the Sc-free sample contains cloudy spots that are responsible for the surface opacity. The formation of those cloudy spots is controlled by the solubility of the atoms in the solid solution. A detailed discussion of this phenomenon is provided later in the text.

**Figure 1:**
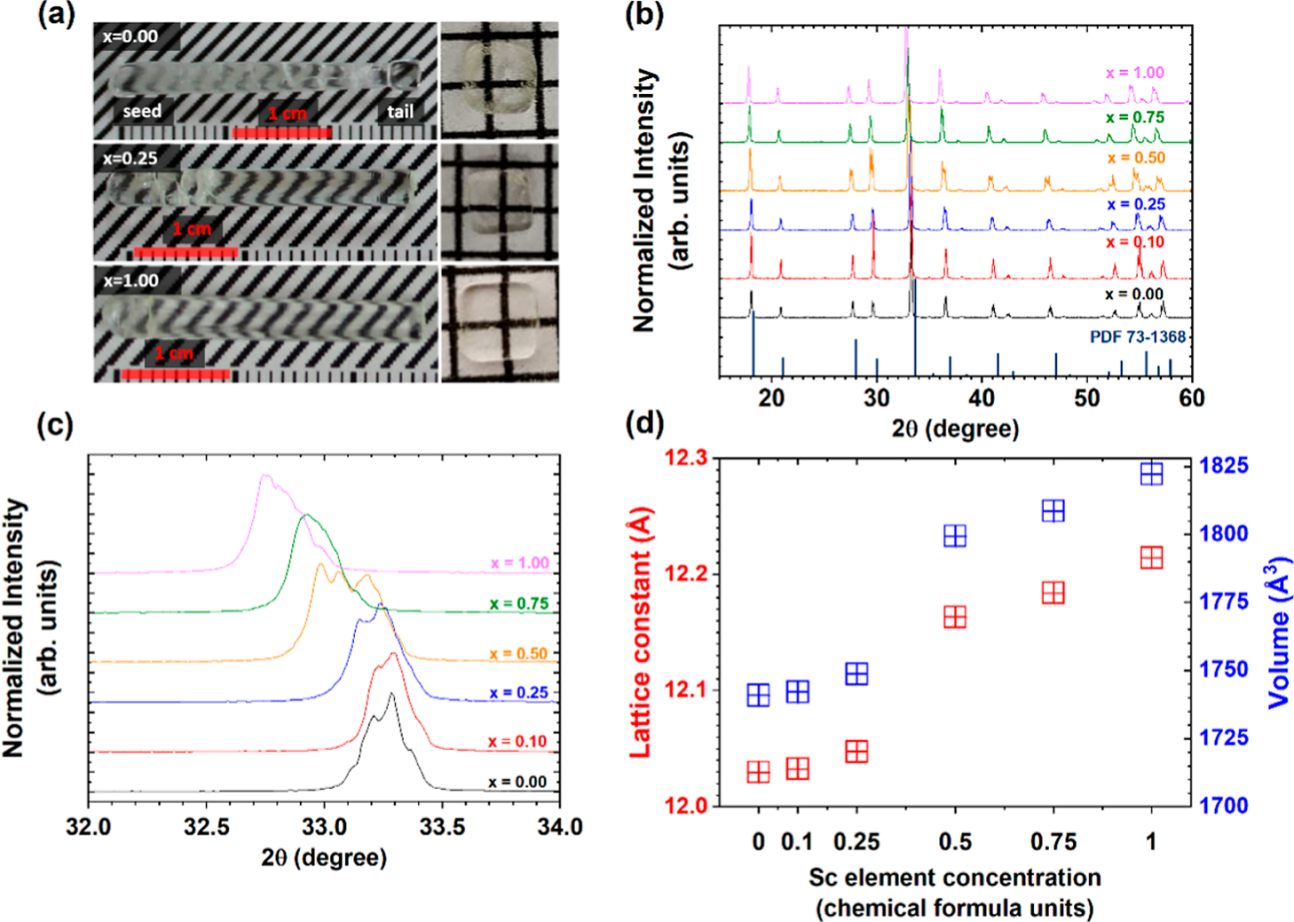
(a) As-grown rods of Pr^3+^-doped Lu_3_Al_2.5–*x*_Sc_*x*_Ga_2.5_O_12_ (where *x* = 0.00, 0.25, 1.00) crystals with polished radial plates cut from the central part of the crystal. (b) Theoretical (Lu_3_Al_5_O_12_, #PDF 73-1360) and experimentally recorded PXRD patterns for Pr^3+^-doped Lu_3_Al_2.5–*x*_Sc_*x*_Ga_2.5_O_12_ crystals with increasing Sc^3+^ ions concentration. (c) High-resolution PXRD pattern focused on the 2θ range of 32 to 34°, illustrating the low-angle shift of the primary Bragg reflection; (d) experimentally determined lattice constant *a*_0_ and cell volume *V* as a function of Sc concentration.

Figure 1b shows PXRD patterns for Pr^3+^-doped Lu_3_Al_2.5–*x*_Sc_*x*_Ga_2.5_O_12_ crystals with increasing Sc^3+^ ions concentration. The structural integrity of the garnet crystal lattice is preserved during the systematic substitution of host atoms by Sc, indicating a high degree of structural tolerance to this particular elemental incorporation. All samples belong to the cubic space group *Ia* 3̅ *d* (no. 230). Figure 1c shows a high-resolution PXRD pattern highlighting the 2θ region between 32 and 34°. With an increasing Sc/Al substitution ratio, the diffraction spots shift to the low-angle direction. This change is attributed to the substitution of Al^3+^_VI_ = 0.54 Å and Ga^3+^_VI_ = 0.62 Å by Sc^3+^_VI_ = 0.745 Å in the octahedral sites and Lu^3+^_VIII_ = 0.977 Å by Sc^3+^_VIII_ = 0.87 Å in the dodecahedral sites, respectively.^16^ Some of the diffraction peaks are broadened and consist of several sublines corresponding to reflections from the same family of *hkl* planes. This asymmetric peak shape provides crucial information about the degree of disorder and the potential formation of nanodomains with slightly different lattice parameters. This fine structural detail is essential for understanding the relationship between atomic arrangements and macroscopic properties in disordered systems.^12^ Figure 1d shows the variation of lattice constant *a*_0_ and unit cell volume as a function of Sc concentration. The experimentally estimated lattice constant and unit cell volume grow nonlinearly with an increasing Sc^3+^ ion concentration. The increase in the Sc/Al substitution ratio gives rise to the lattice constant growth from 12.029 to 12.214 Å and the unit cell volume from 1790.40 to 1822.19 Å^3^. The steepest rise in the lattice constant value and unit cell volume takes place for Lu_3_Al_2.5–*x*_Sc_*x*_Ga_2.5_O_12_ crystals in which the concentration of Sc^3+^ ions ranges from 0.25 to 0.50. This indicates that Sc admixing controls the distribution of Ga/Al between the octahedral and tetrahedral sites. Furthermore, the increased Sc concentration drives the incorporation of Sc atoms into the dodecahedral and octahedral sites. The small change in the lattice constant and unit cell volume for the Lu_3_Al_2.5–*x*_Sc_*x*_Ga_2.5_O_12_ crystals, where *x* = 0.00 and 0.25, can suggest that Sc^3+^ ions preferentially enter the dodecahedral coordination rather than the octahedral one.^12^ The substantial variation observed between the compositions corresponding to *x* = 0.25 and *x* = 0.50 suggests that beyond the critical Sc concentration of 0.25, the dodecahedral sites within the crystal lattice become saturated with Sc atoms. Consequently, any additional Sc atoms incorporated into the structure are compelled to occupy octahedral coordination sites, resulting in a significant expansion of the lattice constants.^12^ This alteration in the coordination environment of Sc atoms leads to a concomitant change in the radial distribution of Al, Ga, and Sc atoms within the lattice. The EDS multielemental mapping images and EDS line profile analyses reveal a discernible change in the elemental homogeneity as the concentration of Sc^3+^ ions increases.

Multielemental EDS mapping can be used as an imaging tool to facilitate the identification of the atom distribution within a crystal. The EDS mapping of the Sc, Ga, Al, Lu, and O elements and the corresponding EDS line profiles are shown in Figures 2 and 3, respectively, as well as in Figure S1 in Supporting Information. The EDS analysis revealed radial variations in the number of Sc, Ga, and Al atoms and a homogeneous distribution of the Lu and O elements. The EDS analysis shows different distributions of the Sc, Ga, and Al atoms, particularly in the crystal rim area, where there are stronger signals for Sc and Ga elements and less intense signals for Al. This means that the crystal rim is enriched with larger Sc and Ga atoms and depleted by Al atoms. In contrast, the central region of the crystal exhibits an inverse compositional trend. Consequently, the core manifests an elevated concentration of Al atoms, concomitant with a relative depletion of the Sc and Ga atoms. This heterogeneous distribution of constituent elements across the crystal’s cross-section suggests the occurrence of segregation phenomena during the growth process. As the grown crystals contain only a trace amount of Pr atoms, their detection is obstructed by a large measurement error. Therefore, the distribution of the aforementioned element is not discussed in the EDS analysis. The multielement EDS profile lines give deeper insight into the change of elemental distribution imposed by Sc admixing. The Pr^3+^-doped Lu_3_Al_2.5_Ga_2.5_O_12_ crystal (where *x* = 0) shows a very significant radial gradient in the concentration of Ga and Al atoms. Specifically, the Al atoms have a stronger signal in the core area and less at the crystal rim, while the signal for Ga atoms shows the opposite behavior. With increasing Sc atom concentration, the radial distribution homogeneity of Ga and Al atoms becomes significantly improved. It can be noticed that the distribution of Sc atoms becomes more heterogeneous at high Sc atom concentrations. The radial variation of the Sc, Ga, and Al atoms is the result of a disordered garnet structure consisting of multitype polyhedra, which are the dodecahedron Ln_3_, the octahedron M_2_, and the tetrahedron M_3_.^40,41^ The stoichiometric chemical formula can generally be presented as Ln_3_M_2_M_3_O_12_. The preference of the crystallographic site is mainly determined by the relative size of the central ion because the three polyhedra are quite different in dimensions. In Y_3_Al_2_Al_3_O_12_, the dodecahedron has a volume of approximately 20 Å^3^, while the approximate volumes of the octahedron and the tetrahedron are only 10 and 3 Å^3^, respectively.^41^ Therefore, large ions are expected to reside in large polyhedral-volume sites. However, the structure of the garnet is flexible; hence, the larger cations can enter smaller polyhedra, like in the case of the AD formed by Y replacing Al in octahedral coordination crystallographic site,^42^ or the preferential occupancy of Ga in the tetrahedron rather than the octahedron.^43^ Furthermore, previous research linked polyhedra distortion with the stability of the garnet phase and the radial distribution of elements.^15,34,44^ Therefore, the theory of the distortion of polyhedral fields seems to match the experimental data. Specifically, substituting larger Ga sites for smaller Al sites significantly deforms and expands the volume of octahedra and tetrahedra. Consequently, the volume ratio between LuO_8_ dodecahedra, Ga/AlO_6_ octahedra, and Ga/AlO_4_ tetrahedra increases significantly. This distorts polyhedra and imposes strain on the Ga and Al sublattices. The accumulated strain energy may be relaxed by significant changes in the segregation of Ga and Al atoms.

**Figure 2:**
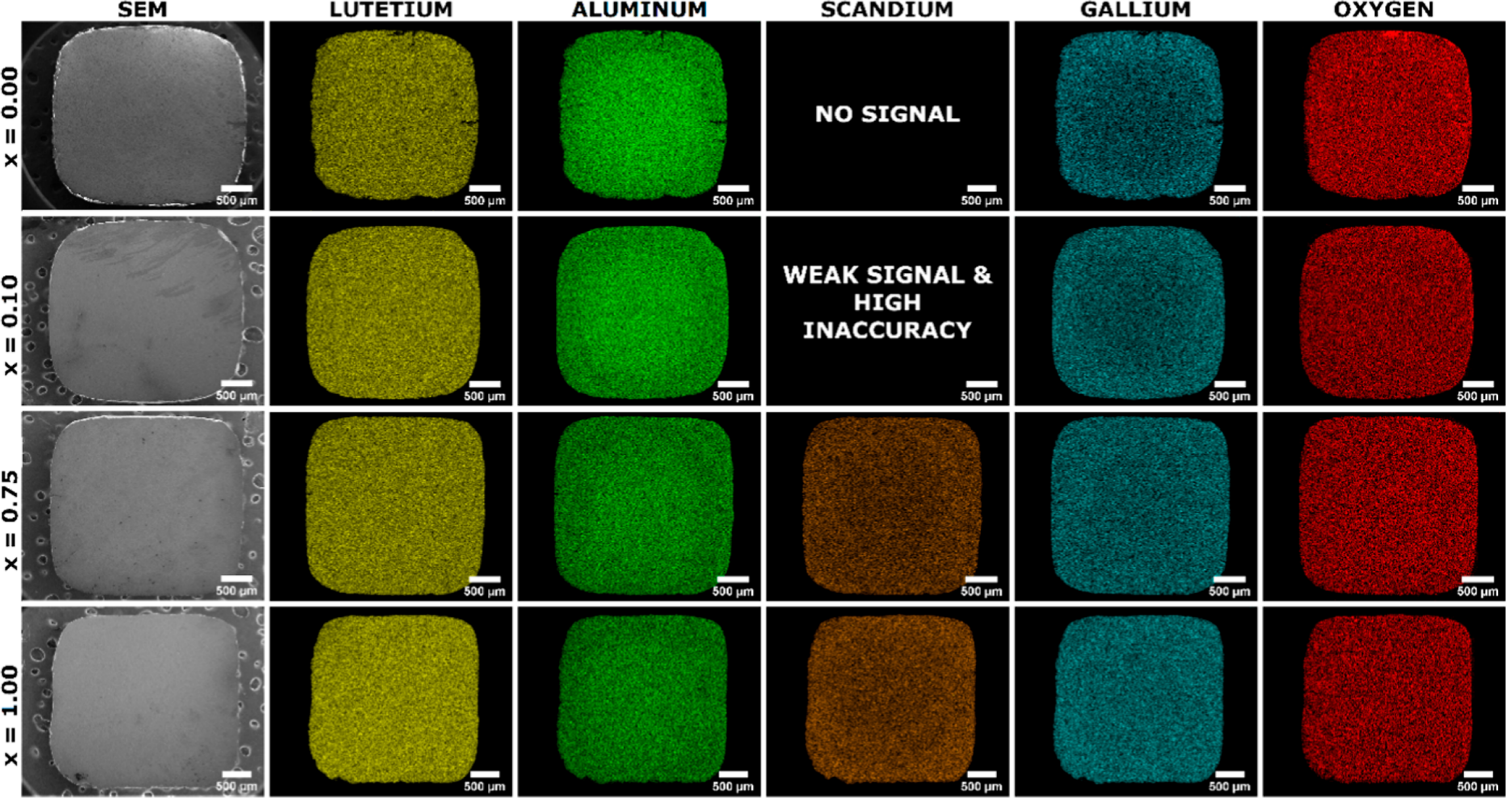
Multielemental EDS mapping of lutetium (Lu, yellow), aluminum (Al, green), scandium (Sc, orange), gallium (Ga, cyan), and oxygen (O, red) of Pr^3+^-doped Lu_3_Al_2.5–*x*_Sc_*x*_Ga_2.5_O_12_ crystals, where *x* = 0.00, 0.10, 0.75, and 1.00.

**Figure 3:**
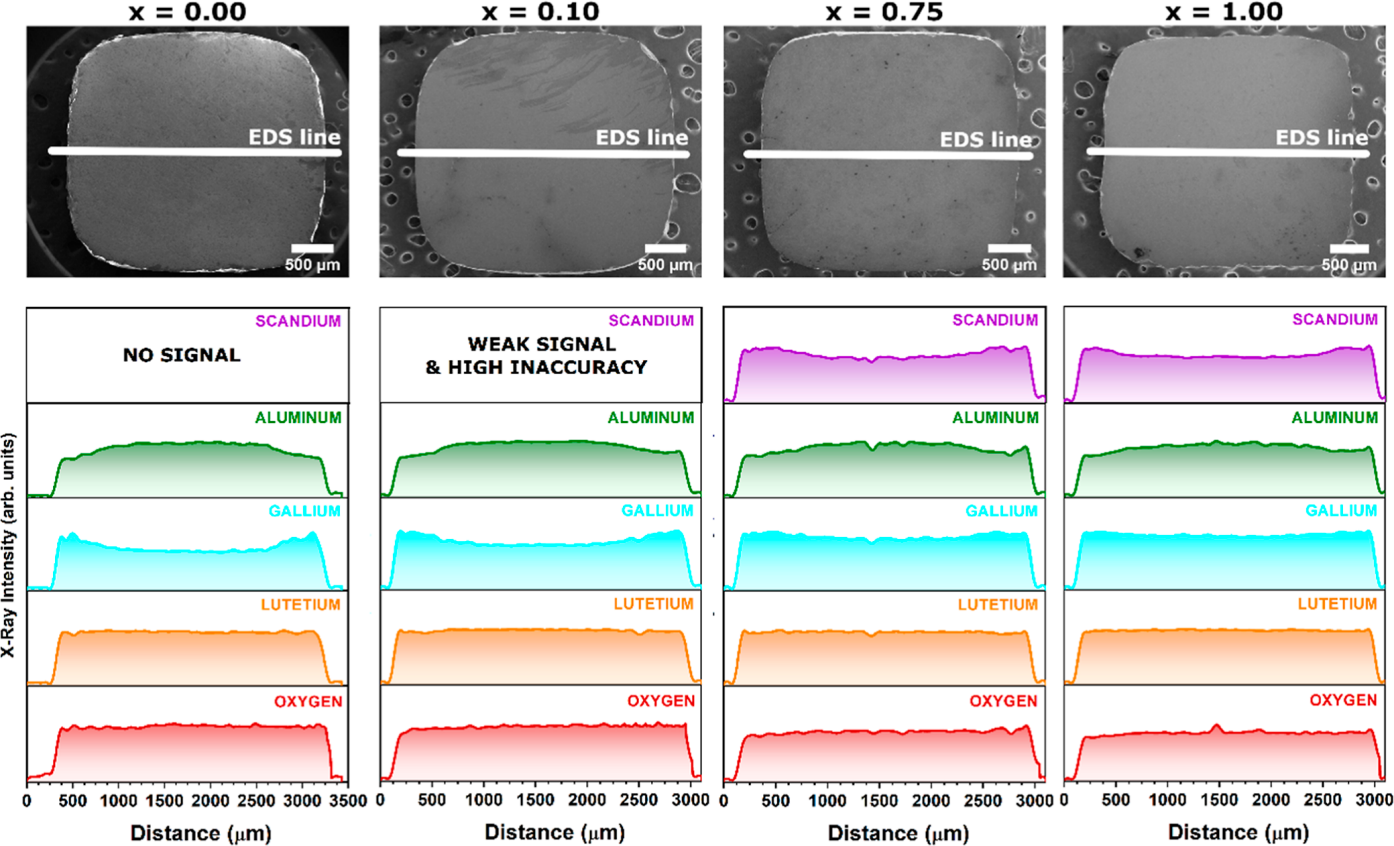
Multielemental EDS line profile of the radial distribution of aluminum (Al, green), scandium (Sc, violet), gallium (Ga, cyan), lutetium (Lu, yellow), and oxygen (O, red) of Pr^3+^-doped Lu_3_Al_2.5–*x*_Sc_*x*_Ga_2.5_O_12_ crystals, where *x* = 0.00, 0.10, 0.75, and 1.00.

The incorporation of mismatched solute elements into a crystal lattice can be enhanced in regions with an increased level of host lattice distortion. For the garnet crystals containing gallium elements, a phenomenon of gallium atom evaporation from the crystal surface was observed, resulting in the formation of surface defects that induce perturbations in the host lattice structure within the proximal regions.^34^ This evaporation process leads to a deviation from the ideal stoichiometry at the surface, thereby disrupting the periodic arrangement of atoms and introducing localized structural distortions within the lattice.^34^ The increased lattice deformation in turn promotes the incorporation of atoms such as Ga and Sc that are not well suited to the host crystal structure.^34^ Furthermore, Marangoni convection occurring in the molten phase at the liquid–solid interface may augment the segregation of Ga and Sc from the crystal rim area. Then, the smaller Al atoms tend to accumulate in the crystal core. The addition of large Sc^3+^_VI_ = 0.745 Å ions^16^ causes the formation of large ScO_6_ octahedra, increasing the crystal strain energy and the volume ratio between the LuO_8_ dodecahedra on the one hand and the Ga/AlO_6_ octahedra and Ga/AlO_4_ tetrahedra on the other hand. To reduce the strain energy and volume ratio of the polyhedra, the largest Sc atoms segregate toward the crystal rim, whereas the distribution of the Ga and Al atoms becomes more uniform. This hypothesis is supported by a previous report for the Gd_3_Al_2_Ga_3_O_12_:Ce crystal, which has revealed a high radial homogeneity of the Al and Ga elements due to the balanced volume ratio between the GdO_8_ dodecahedra, Ga/AlO_6_ octahedra, and Ga/AlO_4_ tetrahedra.^34,45^ Furthermore, this hypothesis is consistent with theoretical calculations of the effects of polyhedral site volume and polyhedra perturbation on the stability of garnet phases,^44^ and with other experimental data.^46^ Importantly, those results demonstrate that Sc incorporation does not compromise the thermodynamic stability of the garnet phase. This experimental finding provides invaluable insights into refining theoretical calculations of the phase diagram for the Al_2_O_3_–Sc_2_O_3_–Ga_2_O_3_–RE_2_O_3_ oxide systems. It is imperative to emphasize that empirical validation through rigorous experimental methodologies is essential for substantiating theoretical approaches and computational models. The integration of experimental evidence remains fundamental for establishing the veracity and reliability of theoretical predictions.

### 3.2 Raman Spectroscopy and High Spatial Resolution PL

The analysis of vibrational spectroscopy as a function of increasing Sc^3+^ ions concentration. The structure of RE_3_M_5_O_12_ RE garnets is assigned to the space group *Ia* 3̅ *d* (O_h_^10^).^47,48^ A primitive cell contains 4 formula units (*Z*_p_ = 4). According to group theory predictions, 25 modes (3A_1g_ + 8E_g_ + 14F_2g_) are Raman-active, and 17 F_1u_ modes are active in the infrared absorption spectrum, while 55 modes (16F_2u_ + 14F_1g_ + 5A_2u_ + 5A_2g_ + 10E_u_ + 5A_1u_) are optically inactive. One F_1u_ mode is acoustic with zero frequency at the Γ point (*q* = 0).^9,47–56^ In general, to perform the assignment of modes in the first-order Raman spectrum of garnet crystals, the experimental spectrum should be analyzed in two regions: low-wavenumber range below 500 cm^–1^ and high-wavenumber range covering 500–900 cm^–1^. The vibrations in the low-wavenumber range are mainly assigned to lattice modes (translational motion of RE^3+^ ions and translational and librational motions of Al/GaO_4_ units) and internal ν_3_ antisymmetric stretching vibration of Al/GaO_4_ groups, while in the high-wavenumber range, ν_1_ (breathing mode), ν_2_ (quadruple), and ν_4_ (deformation) internal modes related to the Al/GaO_4_ tetrahedra appear.^47,51,53,54^ The Lu_3_Ga_5_O_12_ (LuGG) single-crystal garnet is a suitable model for interpreting Raman spectra. To support this interpretation, ab initio theoretical calculations were conducted and the contribution of each atom and molecular unit to the appropriate vibrational modes was determined.^53^ Lu^3+^ ion translations predominate in the spectral range below 270 cm^–1^, while Ga^3+^ ions in the octahedral coordination have the largest contribution to vibrations in the 100–400 cm^–1^ spectral range and a minor contribution around 500 cm^–1^. GaO_4_ tetrahedra contributes to lattice vibrations in the spectral range up to 400 cm^–1^ and between 550 and 730 cm^–1^. Oxygen atoms contribute to vibrations both in the low- and high-wavenumber regions.^53,55,56^ The assignment of Raman modes for Lu_3_Ga_5_O_12_ and Lu_3_Al_5_O_12_ is quite similar; however, some changes in the position of modes can be related to the different unit cell volume, which is greater for gallium garnet, and different masses of Al and Ga atoms. Consequently, there is a red shift of the high-wavenumber modes (i.e., wavenumber shifts toward lower values), which results in the overlap of the high- and medium-wavenumber modes in the Raman spectra of the Lu_3_Ga_5_O_12_ crystal.^53,55^ The abovementioned considerations can be the basis for the interpretation of Raman spectra of the Pr^3+^-doped Lu_3_Al_2.5–*x*_Sc_*x*_Ga_2.5_O_12_ crystals studied in this work.

Figure 4a shows unpolarized Raman spectra of Pr^3+^-doped Lu_3_Al_2.5–*x*_Sc_*x*_Ga_2.5_O_12_ recorded at 300 K using a 514.5 nm laser line in the 100–900 cm^–1^ spectral range. Due to the complexity of that garnet structure, the assignment of Raman modes is rather an ambiguous task. The following is proposed as a tentative assignment: the Raman modes in the 100–420 cm^–1^ spectral range are attributed to the translations of the Lu^3+^ ion, as well as to the translations, librations, and antisymmetric stretching vibrations of AlO_4_ and GaO_4_ tetrahedra, modes in the range between 500 and 580 cm^–1^ are assigned to the bending vibrations of AlO_4_ and GaO_4_ tetrahedra, modes in the 580–700 cm^–1^ spectral range are assigned to the symmetric stretching vibrations of AlO_4_ and GaO_4_ tetrahedra, and modes in the region from 700 to 900 cm^–1^ are attributed to the bending vibrations of AlO_4_ and GaO_4_ tetrahedral units.^52–55^ The number of experimentally observed modes is lower than that predicted by the group theory. This is probably due to the degeneracy of several modes and some modes being too weak to be observed experimentally. The positions and symmetries of the modes observed in the spectra for Pr^3+^-doped Lu_3_Al_2.5–*x*_Sc_*x*_Ga_2.5_O_12_ crystals with *x* = 0,00, 0.50, 1.00 are listed in Table S1 in Supporting Information. As observed in Figure 4a, the changes in the Raman spectra with the increase of the Sc^3+^ ions concentration are noticeable. In particular, the band recorded in the 750–815 cm^–1^ spectral range changes its position significantly. For instance, the position of the maximum of the abovementioned band was recorded for Pr^3+^-doped Lu_3_Al_2.5_Ga_2.5_O_12_ (where *x* = 0.00), Lu_3_Al_2.0_Sc_0.5_Ga_2.5_O_12_ (*x* = 0.50), and Lu_3_Al_1.5_Sc_1_Ga_2.5_O_12_ (*x* = 1.00) crystals at 786, 776, and 773 cm^–1^, respectively. The observed red-shift of this band with the increase in Sc^3+^ ions concentration correlates with the increase in the lattice constant, as depicted in Figure 1d. This band is assigned to the ν_4_ deformation vibration of tetrahedral units (refer to Table S1 in Supporting Information). Figure 4b,c shows the red-shift of the ν_4_ deformation vibration wavenumber as the concentration of Sc^3+^ ions increases. This phenomenon can be attributed to changes in the lattice constant, which, in turn, impact the bond lengths within the tetrahedral units. Specifically, the incorporation of larger Sc^3+^ ions into the lattice causes an expansion of the lattice constant and unit cell volume. Consequently, the observed red-shift of the ν_4_ deformation vibration provides further evidence that the bond lengths within the MO_4_ tetrahedra (where M = Al, Ga) increase significantly due to the Sc-induced expansion of the lattice constant. This finding underscores the influence of Sc substitution on the change of the local structure, elemental distribution, and vibrational properties of the material. Furthermore, Raman-active vibrations that occur in the range up to 300 cm^–1^ in both Lu_3_Al_5_O_12_ and Lu_3_Ga_5_O_12_ garnets are characterized by higher intensity than that observed for Pr^3+^-doped Lu_3_Al_2.5–*x*_Sc_*x*_Ga_2.5_O_12_ crystals. It can be concluded that the presence of three types of octahedra (AlO_6_, GaO_6_, ScO_6_), two types of tetrahedra (AlO_4_, GaO_4_), and two types of dodecahedra (LuO_8_, ScO_8_) in the crystal structure reduces the symmetry of the crystal and increases disorder of the crystal structure, which may reduce the intensity of modes in the low-wavenumber range and worsen the separation of individual bands. As shown below, the distortion of the crystal structures is also reflected in the high-resolution absorption, PL, and radioluminescence spectra (Figures S2, S3, and S5 in Supporting Information).

**Figure 4:**
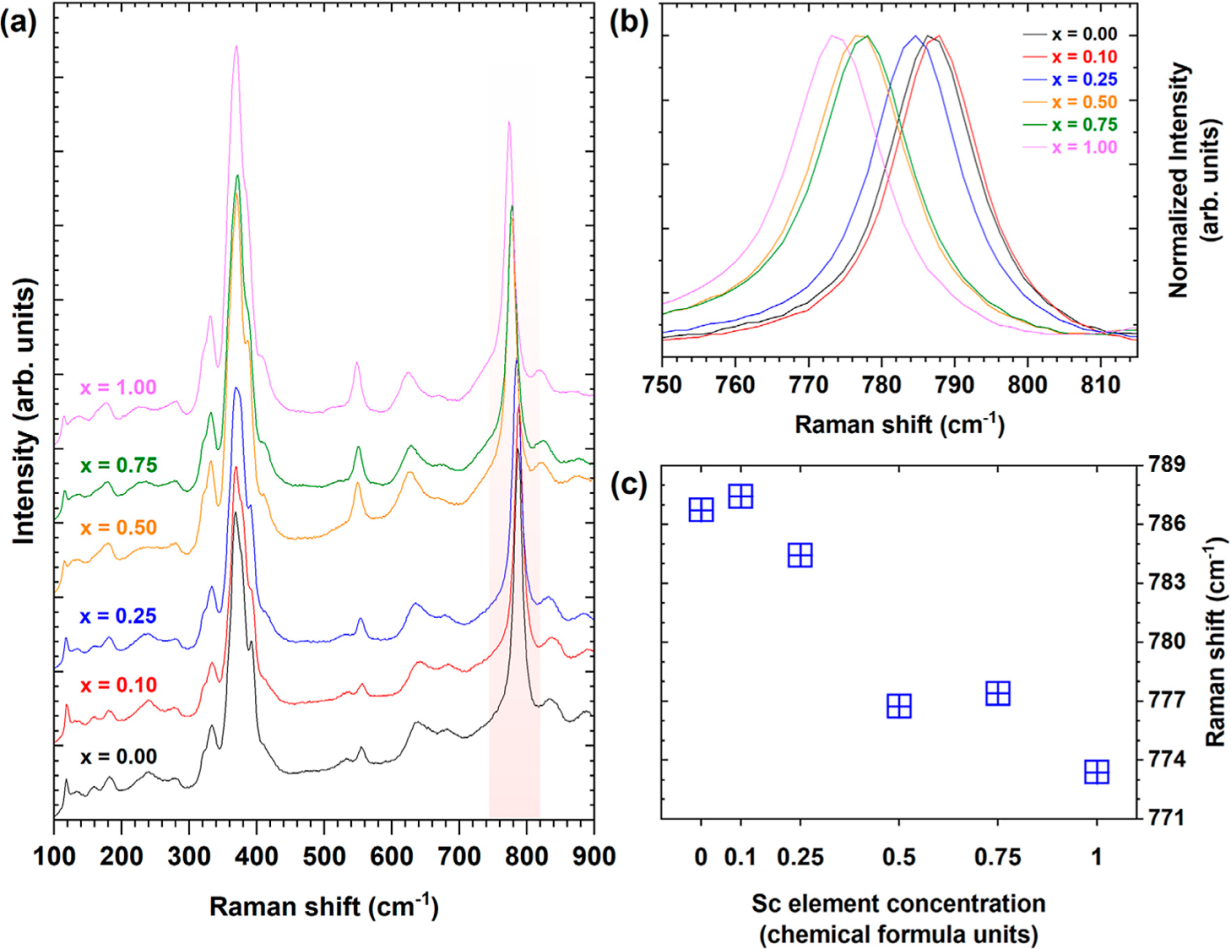
(a) Unpolarized Raman spectra recorded under excitation at 514.5 nm. (b,c) Dependence of the position of the Raman bands recorded in the 750–800 cm^–1^ spectral range as a function of Sc^3+^ ions concentration in Pr^3+^-doped Lu_3_Al_2.5–*x*_Sc_*x*_Ga_2.5_O_12_ crystals.

### 3.3 PL Properties under Synchrotron Radiation Excitation

The absorption spectra of Pr^3+^-doped Lu_3_Al_2.5–*x*_Sc_*x*_Ga_2.5_O_12_ single crystals exhibit characteristic broad bands, attributed to the electronic transitions of the Pr^3+^ ions. These bands, centered at approximately 245 and 275 nm, correspond to the 4f^2^ → 5d_2_^1^4f^1^ and 4f^2^ → 5d_1_^1^4f^1^ absorption transitions, respectively.^33^ A detailed depiction of the absorption spectra is provided in Figure S2 of the Supporting Information.

Figure 5 illustrates the excitation spectra of the Pr^3+^-doped Lu_3_Al_2.5–*x*_Sc_*x*_Ga_2.5_O_12_ crystals for *x* ranging from 0.00 to 1.00 monitored at 299 nm (attributed to the 4f^1^5d_1_^1^ → 4f^2^ transition of Pr^3+^ ions). The spectrum of the Pr^3+^-doped Lu_3_Al_2.5_Ga_2.5_O_12_ crystal (where *x* = 0.00) reveals two distinct excitation bands, 4f^2^ → 5d_1_^1^4f^1^ and 4f^2^ → 5d_2_^1^4f^1^, centered at 4.43 eV (280 nm) and 5.07 eV (245 nm), respectively. The 4f^2^ → 5d_2_^1^4f^1^ excitation band undergoes a shift toward lower energy with increasing Sc^3+^ ions concentration, while the 4f^2^ → 5d_1_^1^4f^1^ excitation band moves toward higher energy. This observation aligns well with a general trend attributed to the decreasing crystal field splitting caused by the larger scandium atoms in the crystal lattice.^57^ The onset of the host-related excitation also experiences a monotonic shift from 6.08 eV (204 nm) to 5.88 eV (211 nm) with increasing Sc^3+^ ions concentration. The onset of host excitation was approximated by identifying the energy corresponding to a half value of the maximum intensity on the slope of the host excitation spectrum above 5.8 eV. The substitution of Sc into an Al site in the Pr^3+^-doped Lu_3_Al_2.5–*x*_Sc_*x*_Ga_2.5_O_12_ crystal lattice induces significant changes in the local environment of the site due to their different ionic radii and electronic configurations. This results in lattice distortions, modification of the crystal field strength, and the formation of localized electronic states. At high Sc element admixing levels, these effects alter the band structure by modulating the conduction band bottom and reducing the band gap width, as discussed by Spassky et al.^58^ These results are consistent with SEM–EDS analysis, as well as absorption and Raman data, providing further evidence that Sc element admixing significantly enhances disorder in the host lattice and provides centers for binding excitonic states. Moreover, synchrotron radiation experiments provide crucial information regarding the role of Sc in the host band gap energy tuning. This knowledge holds potential for the design of novel scintillators and phosphors with tailored band gap energies to meet specific application demands. The anomalous feature observed at approximately 4.55 eV in Figure 5 represents an instrumental artifact.

**Figure 5:**
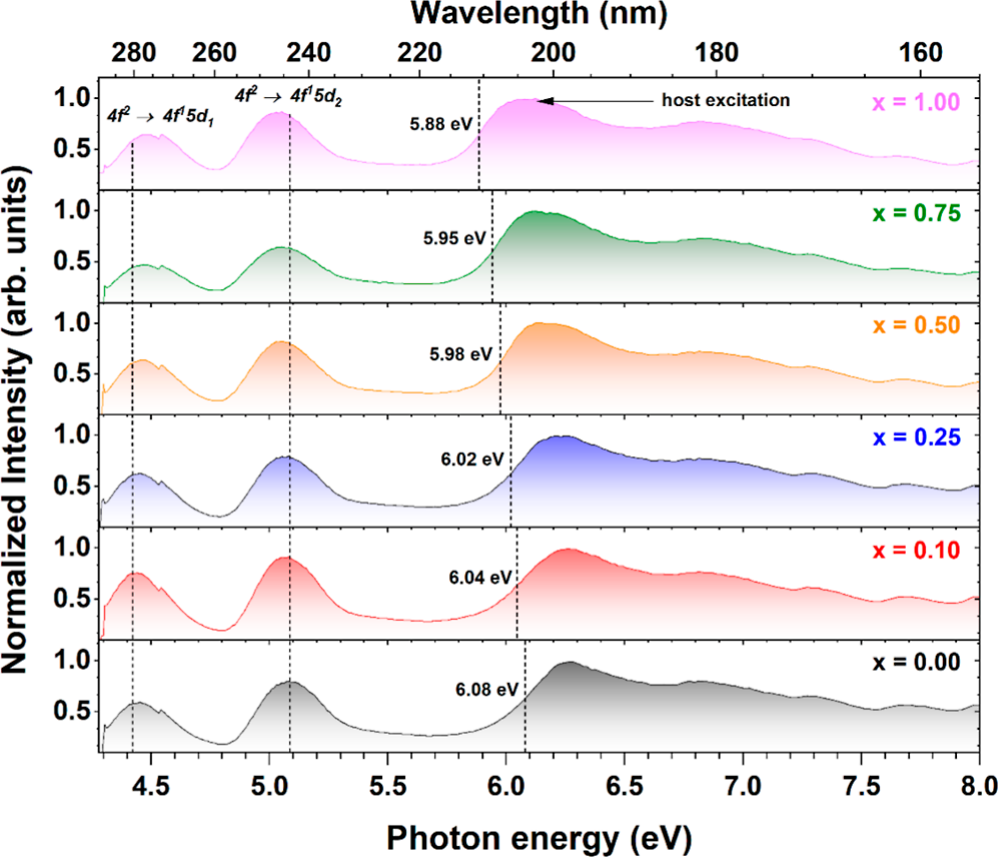
Excitation spectra of Pr^3+^-doped Lu_3_Al_2.5–*x*_Sc_*x*_Ga_2.5_O_12_ crystals with increasing Sc^3+^ ions concentration recorded under UV-VUV excitation at the P66 beamline (λ_emi_ = 299 nm, 300 K).

Figure 6a,b compares the RT emission spectra obtained under the excitation at 7.0 eV (177 nm), 4f^2^ → 5d_2_^1^4f^1^ and 4f^2^ → 5d_1_^1^4f^1^ interconfigurational transitions of Pr^3+^ ions at 5.02 eV (245 nm) and 4.45 eV (278 nm). These spectra comprise the following: (i) emission bands, observed in the 280–420 nm spectral range assigned to the 4f^1^5d_1_^1^ → 4f^2^ interconfigurational transition; and (ii) well-separated and relatively intense emission lines originating from the relaxation of the ^3^P_0_ and ^1^D_2_ excited states of Pr^3+^ ions, observed in the visible spectral range (480–750 nm). Incorporating Sc elements into the Pr^3+^-doped Lu_3_Al_2.5–*x*_Sc_*x*_Ga_2.5_O_12_ crystal lattice significantly alters its luminescence properties. This phenomenon is attributed to the interplay of crystal field effects and lattice disorders induced by Sc admixing. These factors influence the excited state dynamics of Pr^3+^ ions, promoting the 5d_1_^1^ → ^3^P_*J*_ crossover pathway and enhancing the emission originating to the 4f^2^ → 4f^2^ intraconfigurational transitions, while concurrently quenching the emission from the 4f^1^5d_1_^1^ → 4f^2^ interconfigurational transition typically observed at 300 K. Notably, the thermal ionization of the 5d_1_^1^ excited state of Pr^3+^ ion further contributes to the observed quenching effect.^5,15,59–61^ It is worth mentioning that under the interband excitation, Tb^3+^ ions trace impurities could also be detected in the 383–450 and 500–650 nm spectral regions.^62–64^ Figure 6c illustrates the correlation between maximum emission peak intensities in the 300–305 nm spectral range and increasing Sc concentration, measured at 12.6 and 293 K temperatures under interband excitation at 7.0 eV. Conversely, Figure 6d depicts the relationship between maximum emission peak intensities within the same spectral range (300–305 nm) and increasing Sc concentration, under excitation into the 4f^2^ → 5d_2_^1^4f^1^ and 4f^2^ → 5d_1_^1^4f^1^ interconfigurational transitions of Pr^3+^ ions at 5.02 and 4.45 eV at 12.6 and 293 K.

**Figure 6:**
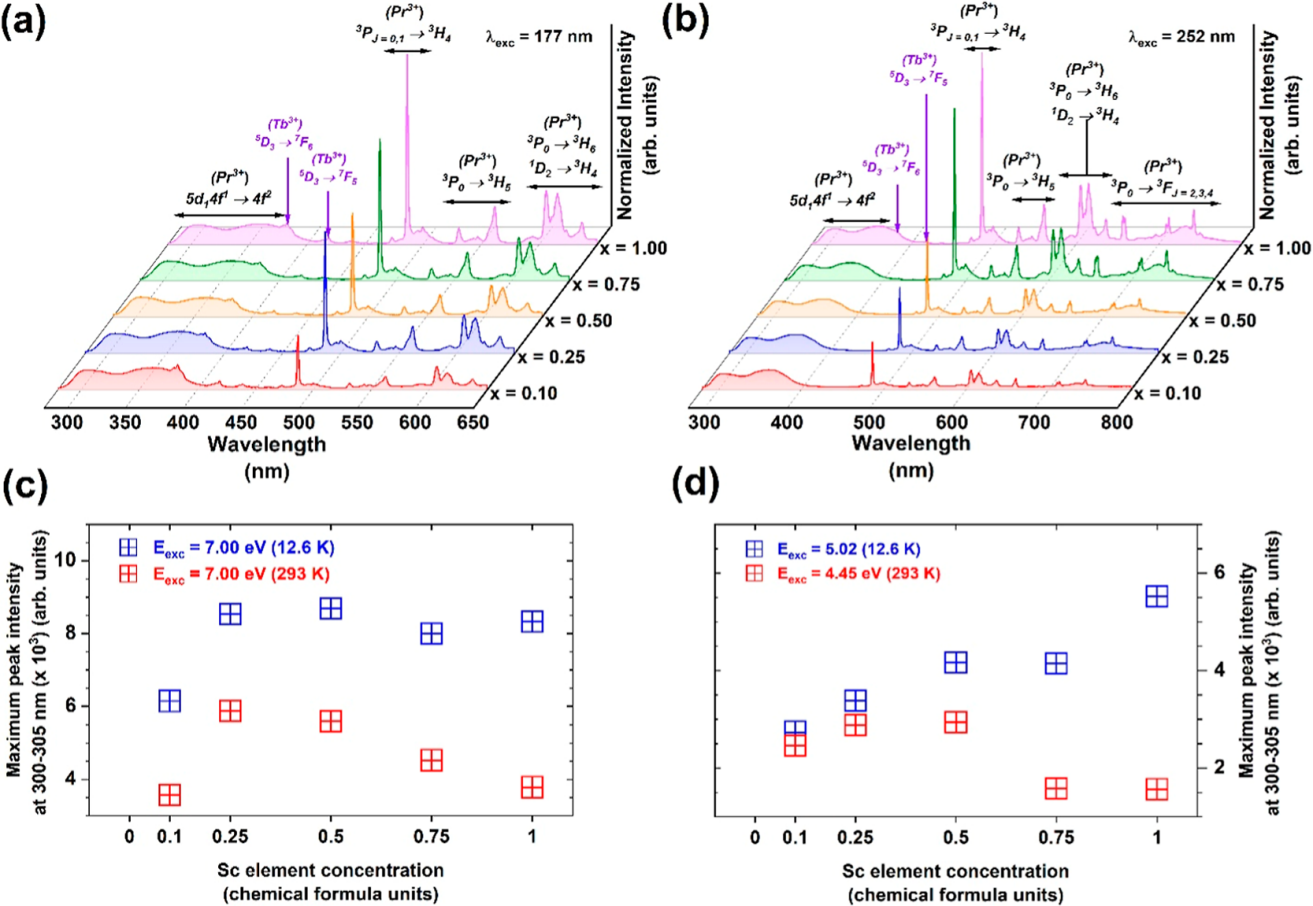
Emission spectra of Pr^3+^-doped Lu_3_Al_2.5–*x*_Sc_*x*_Ga_2.5_O_12_ crystals with increasing Sc^3+^ ions concentration under excitation at (a) 7.0 eV (177 nm, RT) and (b) 4.45 eV (278 nm, RT). The maximum emission peak intensity in the 300–305 nm spectral range is observed at temperatures of 12.6 and 293 K, resulting from (c) interband excitation at 7.0 eV, and (d) interconfigurational 4f^2^ → 5d_2_^1^4f^1^ excitation at energies of 5.02 eV and 4f^2^ → 5d_1_^1^4f^1^ 4.45 eV. The measurements were conducted at the P66 beamline.

Under interband (7.0 eV) and 4f^2^ → 5d_2_^1^4f^1^ interconfigurational transitions of Pr^3+^ ions at 5.02 eV excitations at 12.6 K, the Pr^3+^ 4f^1^5d_1_^1^→ 4f^2^ emission intensity shows a nonlinear enhancement with increasing Sc^3+^ ions concentration. The observed enhancement in emission intensity can be attributed to the increased concentration and more uniform distribution of Pr^3+^ ions achieved with a higher Sc concentration (see Figure S4). As a result, the probability of Pr^3+^ emission quenching due to the Pr^3+^–Pr^3+^ pairs is significantly reduced. The increase in Sc^3+^ ions concentration can also impact the energy transfer from the host lattice to Pr^3+^ ions. In addition, the 4f^1^5d_1_^1^ → 4f^2^ interconfigurational transitions in Pr^3+^ ions are first-order, orbitally electric dipole-allowed, resulting in high radiative transition probabilities. The enhancement of Pr^3+^ 4f^1^5d_1_^1^ → 4f^2^ emission intensity can be attributed to the incorporation of Sc^3+^ ions, which improves energy transfer to Pr^3+^ centers and simultaneously reduces the influence of quenching channels that deteriorate luminescence performance. This observation is corroborated by the dependence of the maximum peak intensity on Sc^3+^ ions concentration under interband excitation at 7.0 eV at 293 K (Figure 6c). The maximum intensity of the 4f^1^5d_1_^1^ → 4f^2^ transition is highest for the Pr^3+^-doped Lu_3_Al_2.25_Sc_0.25_Ga_2.5_O_12_ crystal (where *x* = 0.25) and gradually diminishes with further Sc^3+^ ions concentration increase due to luminescence quenching processes. Nonetheless, even for the Pr^3+^-doped Lu_3_Al_1.5_Sc_1.0_Ga_2.5_O_12_ crystal (where *x* = 1.00), the intensity remains higher than that for the Pr^3+^-doped Lu_3_Al_2.4_Sc_0.1_Ga_2.5_O_12_ crystal (*x* = 0.10). This finding indicates that the efficiency of energy transfer from the host lattice to Pr^3+^ ions is more efficient than the emission quenching mechanism assigned to the 4f^1^5d_1_^1^ → 4f^2^ transition of Pr^3+^ ions. This conclusion aligns with the dependence of the maximum peak intensity on Sc^3+^ ions concentration under excitation at 4.45 eV at 293 K (Figure 6d). The maximum intensity initially ascends for the Pr^3+^-doped Lu_3_Al_1.75_Sc_0.25_Ga_2.5_O_12_ crystal (*x* = 0.25) and subsequently declines with further increasing Sc^3+^ ions concentration. Notably, owing to the absence of energy transfer from the host lattice to Pr^3+^ ions at this excitation (4.45 eV, 293 K), the emission intensity between 300 and 305 nm (depending on Sc^3+^ ions concentration) for the Pr^3+^-doped Lu_3_Al_2.5–*x*_Sc_*x*_Ga_2.5_O_12_ crystals (where *x* = 0.75 and 1.00) is lower than that for the Pr^3+^-doped Lu_3_Al_2.4_Sc_0.1_Ga_2.5_O_12_ crystal (*x* = 0.1), a stark contrast to the interband excitation process (7.0 eV). This observation reinforces the notion that Sc elements enhance both the energy transfer from the host lattice to Pr^3+^ ions and the uniformity of the Pr^3+^ ion distribution. On the other hand, Sc admixing induces both lattice perturbations and a reduction in the energy barrier between the CBM and the 5d_1_^1^ excited state of the Pr^3+^ ions. These factors synergistically create a nonradiative quenching pathway for the Pr^3+^ ions emission: the ionization of the 5d_1_^1^ excited state and the 5d_1_^1^ to ^3^P_*J*_ crossover flow of the excited electron,^5,15,59–61^ causing the appearance of shorter decay components originating from the crystal areas with higher Sc^3+^ ions concentration. This quenching effect is prominent at elevated temperatures (as observed in Figure 6c,d at 293 K), leading to a shift in emission intensity toward the 4f^2^ → 4f^2^ intraconfigurational transition of Pr^3+^ ions.

The observed maximum intensity in Pr^3+^-doped Lu_3_Al_2.5_Sc_*x*_Ga_2.5_O_12_ (where *x* = 0.25) crystal can be attributed to an optimal balance between enhanced energy transfer and minimized nonradiative quenching mechanisms. At this Sc concentration, the incorporation of Sc^3+^ ions alters the local crystal field environment and increases the lattice disorder. This modification leads to a more uniform distribution of Pr^3+^ ions, thereby reducing the likelihood of Pr^3+^–Pr^3+^ pair interactions that can result in emission quenching. This uniformity enhances the efficiency of energy transfer from the host lattice to Pr^3+^ ions, thereby increasing the emission intensity. However, as the Sc^3+^ concentration increases above *x* = 0.25, further lattice perturbations occur, which introduces additional nonradiative pathways. These pathways, such as ionization of the 5d_1_^1^ excited state or crossover transitions to the ^3^P_*J*_ states, become more prominent, especially at elevated temperatures. Consequently, the emission intensity diminishes due to these quenching mechanisms. The variation in trends with temperature is indicative of the temperature dependence of these radiative and nonradiative processes. At 12.6 K, nonradiative quenching mechanisms are less active, allowing the enhanced energy transfer and reduced Pr^3+^–Pr^3+^ quenching at higher Sc^3+^ concentrations to dominate, leading to increased emission intensity. In contrast, at 293 K, nonradiative processes become more significant, counteracting the benefits of increased Sc^3+^ concentration and resulting in a decrease in emission intensity. The observed changes in emission intensities with increasing Sc^3+^ ion concentration provide evidence of the interplay between crystal field effects and lattice disorder caused by Sc admixing. This conclusion is further substantiated by the observation of nonexponential decay kinetics in the emission of Pr^3+^, as illustrated in Figure S5 and detailed in Table S2. From the perspective of lattice engineering, Sc elements substitution emerges as a promising approach to increase host lattice disorder, enhancing the absorption strength and emission intensity of 4f^1^5d_1_^1^ → 4f^2^ interconfigurational and 4f^2^ → 4f^2^ intraconfigurational transitions, see Table S3 in Supporting Information.

The consistent PL data confirm that incorporating Sc induces a downward shift in the conduction band minimum, as evidenced by a corresponding low-energy shift in the fundamental absorption edge. This implies a decrease in the band gap energy. Additionally, Sc admixing modifies the local crystal field surrounding Pr^3+^ ions, resulting in a decrease in the energy level splitting of their 5d^1^ states. This is evidenced by the decrease in energy separation between the excitation bands attributed to the 4f^2^ → 5d_1_^1^4f^1^ and 4f^2^ → 5d_2_^1^4f^1^ interconfigurational transitions (see Figure 5). Notably, the downward shift of the CBM and the energy increase of the 5d_1_^1^ state induced by Sc admixing decreases the energy barrier between the CBM and the 5d_1_^1^ state of Pr^3+^ ion, facilitating the escape of an electron from 5d_1_^1^ toward CB (ionization effect).^5,59^ The global concentration of Pr^3+^ ions exhibits a slight variation between samples, increasing with enhanced Sc^3+^ ions concentration (see discussion of Figures S4 and 6). This phenomenon is attributed to the lattice expansion induced by Sc atoms, which augments the solubility of Pr^3+^ ions in the host lattice. Additionally, increasing Sc^3+^ ions concentration promotes improved radial homogeneity of the samples (as can be seen from Figures 2, 3, S4, and 6), which, in turn, enhances the radial homogeneity of Pr^3+^ ions (see Figures S4 and 6). In essence, elevated Sc^3+^ ions concentration fosters a more uniform distribution of Pr^3+^ ions, diminishing clustering or pairing of Pr^3+^ ions, thereby suppressing cross-relaxation processes. This effect is particularly pronounced in the crystal rim region, where Pr^3+^ ions tend to accumulate (as observed in Figure S4). The faster component is suggested to originate from crystal rim areas strongly distorted by Sc admixing, and the slower component, with a decay time closer to that in the sample without Sc^3+^ ions, is suggested to originate from the less perturbed crystal core areas. The contributions of these components to the total 4f^1^5d_1_^1^ → 4f^2^ are strongly correlated to the Sc^3+^ ions concentration and radial distribution of Al, Ga, and Sc atoms in the crystals (see Figures 2 and 3). The same trend was reported in the Y_3_(Al,Ga)_5_O_12_:Pr phosphors, where with increasing Ga concentration, the intensity of the 4f^1^5d_1_^1^ → 4f^2^ transitions decreases and the intensity of the 4f^2^ → 4f^2^ transitions increases.^61,64^

### 3.4 Scintillation Properties

The radioluminescence spectra of the Pr^3+^-doped Lu_3_Al_2.5–*x*_Sc_*x*_Ga_2.5_O_12_ crystals match the trends observed in Figure 6a,b and are presented for detailed reference in Figure S6 of the Supporting Information. The scintillation decay curves and pulse-height spectra of Pr^3+^-doped Lu_3_Al_2.5–*x*_Sc_*x*_Ga_2.5_O_12_ crystals, where *x* = 0.00, 0.10, 0.25, 0.50, 0.75, 1.00 are presented in Figure 7a,b, in Table 1, and Figure S6a,b in Supporting Information. In Sc-containing examined crystals, both the fast and slow scintillation decay time components (Figures 7a and S7a in Supporting Information) exhibit a continuous acceleration with increasing Sc^3+^ ions concentrations. As the Sc^3+^ concentration increases, the fraction of the total intensity (FTI) associated with the fast decay component (τ_1_) steadily increases, from 49% without Sc^3+^ substitution to 67% at *x* = 1.00. Conversely, the FTI of the slow component (τ_2_) decreases, from 51% to 33%, over the same range. An exception to the observed trend is evident in the crystal with a Sc^3+^ concentration of *x* = 0.25, which demonstrates the strongest contribution of the fast scintillation component to the overall scintillation decay process. This notable shift in the balance of the FTI indicates that the structural disorder introduced by Sc^3+^ ion substitution preferentially promotes faster, nonradiative recombination pathways. The incorporation of Sc^3+^ ions into the crystal lattice is associated with lattice distortions in the vicinity of Pr^3+^ ions. These distortions modify the local crystal environment, potentially enhancing the energy transfer efficiency of Pr^3+^ ions in disordered sites. As a result, the probability of transferring excitation energy to trapping centers or defect states is significantly reduced. This mechanism not only accelerates the scintillation decay but also emphasizes the dominance of the fast scintillation component in the decay dynamics. Those observations align well with the findings from PL studies, corroborating the previously proposed mechanisms responsible for the quenching of the 4f^1^5d_1_^1^ → 4f^2^ interconfigurational emission in favor of enhanced 4f^2^ → 4f^2^ intraconfigurational emission intensity.^8,65^ Figures 7b and S7b in the Supporting Information show pulse height spectra measured with a ^137^Cs radioisotope and amplifier shaping time of 2 μs. The pulse-highest spectra demonstrate that increasing Sc^3+^ ions promote the quenching of fast emission attributed to the 4f^1^5d_1_^1^ → 4f^2^ interconfigurational transition of Pr^3+^ ions. Therefore, the LY values decrease significantly with increasing Sc^3+^ ions concentration (see Table 1). Figure 7c presents a comparison of the integrated emission intensities recorded in the 250–450 nm spectral range and the scintillation LY values as a function of increasing Sc^3+^ ions concentration. Both show a consistent decreasing trend, confirming that Sc admixing triggers emission quenching of the Pr^3+^ ions. Moreover, a relatively smaller decrease of RL emission integral compared to LY values suggests that a part of the ionized electrons returns from the CB and radiatively recombines with Pr^4+^ beyond the time gate of LY (delayed recombination luminescence). Figure 8a provides comparative analyses of the general scintillation mechanism in host lattices with varying degrees of structural disorder. Figure 8a illustrates the scintillation process in a host lattice with low disorder, while Figure 8b depicts the mechanism in a highly disordered Sc^3+^-admixed lattice. The introduction of Sc^3+^ ions induces significant modifications in the local crystal field, affecting charge carrier dynamics and energy transfer pathways and thereby influencing the scintillation performance.

**Figure 7:**
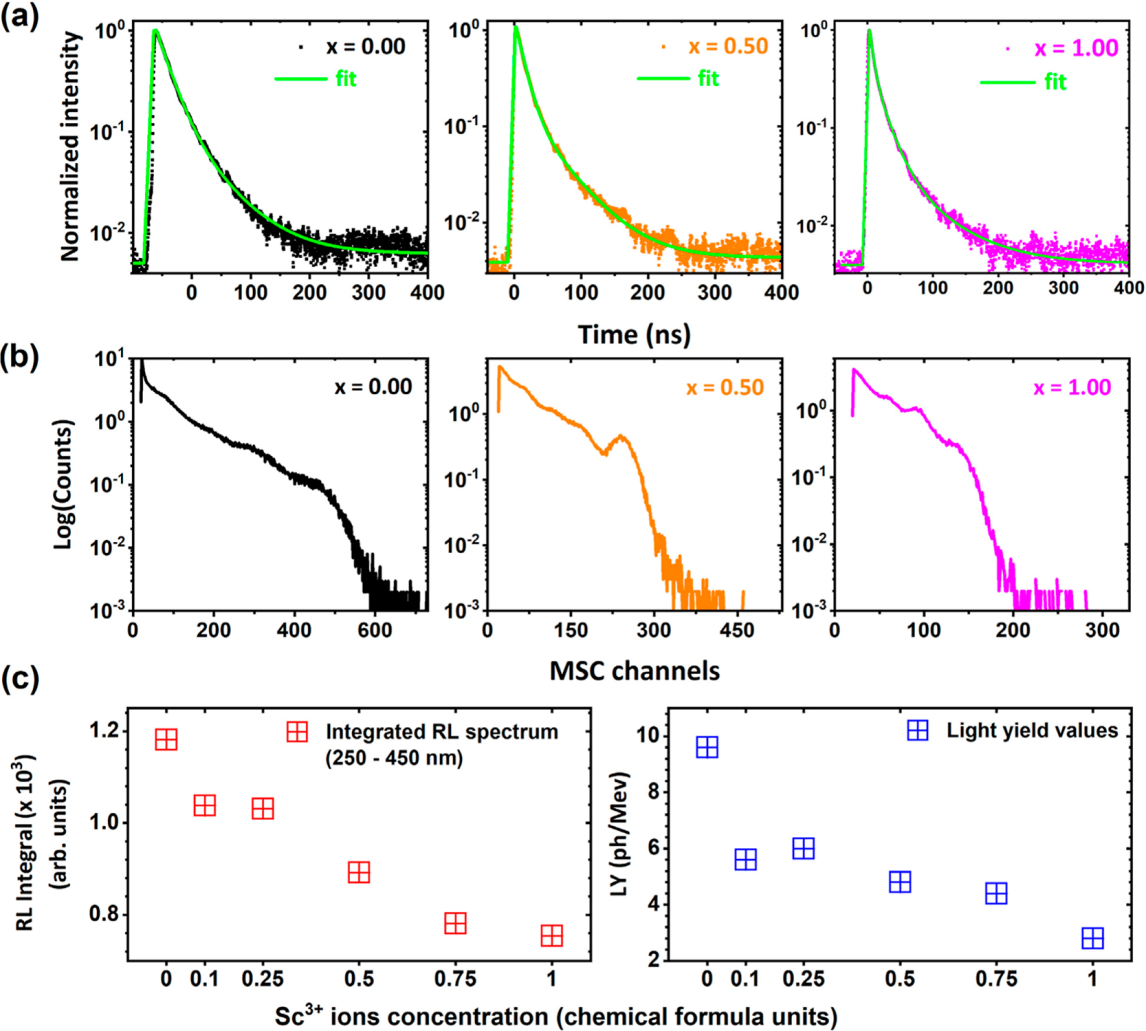
(a) Scintillation decay curves and (b) pulse-height spectra of Pr^3+^-doped Lu_3_Al_2.5–*x*_Sc_*x*_Ga_2.5_O_12_ crystals with increasing Sc^3+^ ions concentration under γ-rays excitation from the ^137^Cs radioisotope. (c) Integrated emission intensities recorded in the 250–450 nm spectral range and scintillation yield values as a function of increasing Sc^3+^ ions concentration.

**Table 1:** Scintillation Decay Times and LY Values (Shaping Time 2 μs) for the Pr^3+^-Doped Lu_3_Al_2.5–*x*_Sc_*x*_Ga_2.5_O_12_ Crystals with Increasing Sc^3+^ Ions Concentration under γ-rays Excitation from ^137^Cs Radioisotope

	scintillation decay times				
Sc^3+^ ions concentration (chemical formula units)	τ_1_ (ns)	FTI of τ_1_ (%)	τ_2_ (ns)	FTI of τ_2_ (%)	LY (photons/MeV) (2 μs)
0.00	22	49	77	51	9600
0.10	10	74	40	26	5200
0.25	8	54	34	46	6000
0.50	8	57	30	43	4800
0.75	6	62	26	38	4400
1.00	5	67	21	33	2800

**Figure 8:**
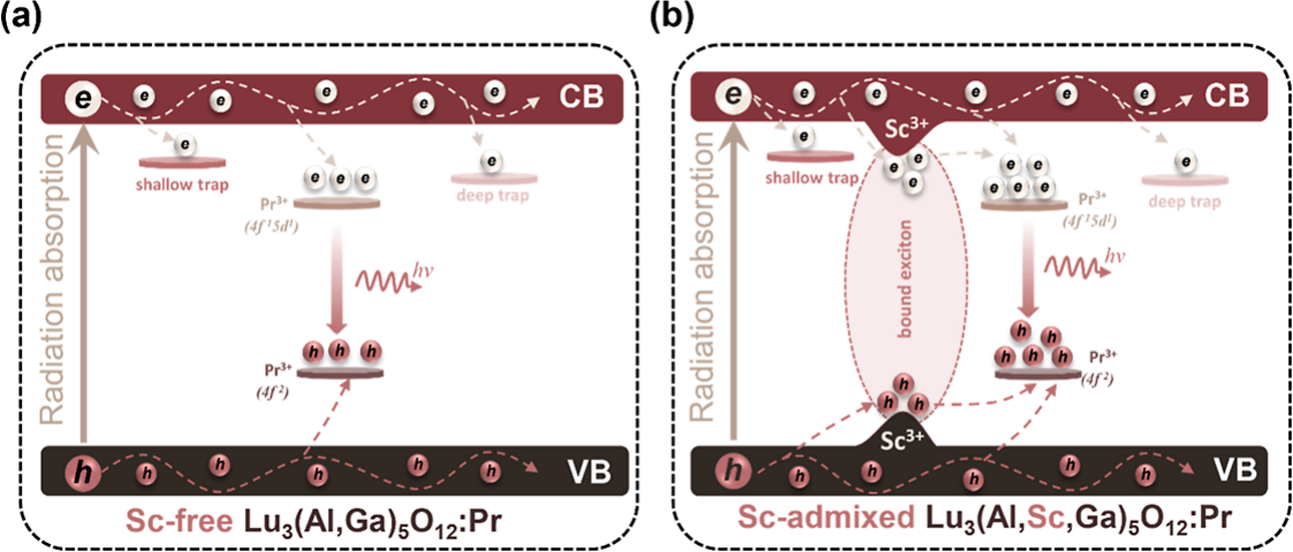
Schematic illustration of the scintillation mechanisms in (a) Sc-free Pr^3+^-doped Lu_3_(Al,Ga)_5_O_12_ and (b) Sc-admixed Pr^3+^-doped Lu_3_(Al,Sc,Ga)_5_O_12_ crystals. In the Sc-admixed system, additional excitons bound at Sc^3+^ sites transfer energy to Pr^3+^ altering the carrier capture pathways compared to the Sc-free host.

As illustrated in Figures 8b, the interaction of X-ray radiation with the material leads to the generation of free electrons and holes. These charge carriers may subsequently be captured either by Pr^3+^ ions or by structural trapping centers, including shallow and deep traps.^66,67^ In the Sc-admixed crystal, the incorporation of Sc^3+^ ions introduces localized perturbations in the electrostatic potential, facilitating the formation of bound excitonic states. The strong resonance between these excitons localized at Sc^3+^ sites and the energy states of Pr^3+^ ions enables the transfer of energy from Sc^3+^ to Pr^3+^ ions. The incorporation of Sc atoms exerts a minimal effect on the modification of trapping center properties. Specifically, the influence of both shallow and deep traps on the scintillation characteristics remains marginal; see Figure S7 and Tables S4 and S5 in Supporting Information. This suggests that the primary mechanisms governing the scintillation and luminescence properties, tuned by Sc admixing, are predominantly associated with the energy transfer from Sc^3+^ ions to Pr^3+^ ions, as well as the reduction in local symmetry. Furthermore, the enhanced rate of the 5d_1_ → ^3^P_*J*_ crossover of excited electrons plays a crucial role in determining the luminescence dynamics.

## 4 Conclusions

This study significantly advanced the understanding of Sc-admixed garnet crystals, addressing the very limited research data on the influence of Sc atoms in such systems. The successful crystallization of Pr^3+^-doped Lu_3_Al_2.5–*x*_Sc_*x*_Ga_2.5_O_12_ crystals via the micropulling down method, despite the considerable atomic radius mismatch between Sc^3+^ (*r*_VI_ = 0.745 Å) and Al^3+^ (*r*_VI_ = 0.535 Å), provided valuable insights into the structural accommodation of Sc atoms within the garnet lattice. These findings substantially expanded our understanding of the role of Sc^3+^ ions as an alternative substituent for Al^3+^ and Ga^3+^ ions in mixed garnets, contributing to the fundamental knowledge of crystal chemistry in complex oxide systems and opening new possibilities for tailoring structural, luminescence, and scintillation properties of garnet systems. Importantly, this findings demonstrated that Sc^3+^ ions incorporation did not compromise the thermodynamic stability of the garnet phase, providing invaluable experimental data to refine theoretical calculations of the phase diagram for Al_2_O_3_–Sc_2_O_3_–Ga_2_O_3_-RE_2_O_3_ oxide systems.

The introduction of Sc^3+^ ions into the garnet lattice resulted in significant structural and electronic modifications, which influenced the luminescence and scintillation properties of the crystals. XRD analysis confirmed that the garnet structure remained stable, while the lattice constants and cell volumes increased nonlinearly with Sc^3+^ ions concentration, attributed to the expansion of polyhedral volume and the relaxation of strain energy induced by Sc^3+^ ions. Energy-dispersive X-ray spectroscopy mapping revealed an inhomogeneous spatial distribution of constituent elements, characterized by elevated concentrations of Sc and Ga elements at the crystal rim with Al predominantly concentrated in the core region. The increased Sc^3+^ ions content significantly improved the compositional uniformity and improved the radial distribution of Pr^3+^ ions throughout the crystal volume. The observed broadening and shifting of Raman bands provided evidence of lattice disorder, correlating with increased Sc concentration. Such structural disorder directly influenced the crystal field strength and altered the position of the Pr^3+^ ions 5d_1_^1^ excited state relative to the conduction band minimum. Notably, Sc^3+^ ions incorporation reduced local symmetry around Pr^3+^ ions, thereby lowering the energy barrier between the 5d_1_^1^ excited state and the lower-lying 4f energy state. This reduction facilitated nonradiative 5d_1_^1^ to ^3^P_*J*_ crossover of excited electrons and ionization of the 5d_1_^1^ excited state. Consequently, emission intensity shifted toward the 4f^2^ → 4f^2^ interconfigurational transitions. The reduction in local symmetry and the enhanced rate of 5d_1_^1^ to ^3^P_*J*_ crossover of excited electrons led to a decrease in the 4f^1^5d_1_^1^ → 4f^2^ interconfigurational scintillation LY while simultaneously accelerating the scintillation response. This inverse relationship between LY and response time is a common characteristic in scintillator materials, where modifications that speed up the response often result in the reduced light output. The synchrotron radiation experiments provided deep insights into the role of Sc^3+^ ions in tuning the band gap energy, presenting opportunities for tailoring scintillators and phosphors for specific applications. Thermoluminescence glow curve analysis identified trapping centers linked to antisite Lu^*x*^_Al_ dislocations below 200 K and oxygen vacancies (V_O_^••^) combined with other centers above 280 K. Despite their presence, these defects exerted minimal influence on the scintillation efficiency. The deconvolution of the TL glow curves into single peaks revealed that Sc admixing reduced the number of distinct energy levels by merging separate peaks (at 380 and 322 K) into a single maximum (375–353 K). The trap depths ranged from 1.63 eV (deep traps) to 0.22 eV (shallow traps) across all samples, with frequency factors predominantly between 10^7^ and 10^11^ s^–1^, consistent with first-order thermoluminescent kinetics. The observed resilience of the Sc-admixed system against the formation of shallow and deep trapping centers is a finding of significant importance for the design of oxide scintillators. Furthermore, the modulation of band gap energy through Sc incorporation is consistent with studies demonstrating that such admixtures can effectively tune structural and luminescence properties, enhancing the material suitability for targeted applications.

## Supplementary Material



## Notes

Crystal phase and morphology: EDS mapping and radial line profiles; Raman spectroscopy characteristics: experimental wavenumbers and mode assignments; absorption and PL properties: absorption spectra; PL maps; high spatial-resolution PL spectra; emission band positions and fwhm comparisons; PL decay kinetics: PL decay curves; decay time values; scintillation properties: radioluminescence spectra; scintillation decay curves and pulse-height spectra; thermally stimulated luminescence: TL glow curves; trap parameters (peak temperatures, activation energies, frequency factors); and peak temperatures, activation energies (PDF)
