# Supplementary material for: Correlating Structural Disorder and Pr3+ Emission Dynamics in Lu3Al2.5–x Sc x Ga2.5O12 Crystals: A Comprehensive Structure–Property Investigation
Source: ACS Omega. 2025 May 8;10(19):19817–31. doi: 10.1021/acsomega.5c01062 (PMC12096190; doi:10.1021/acsomega.5c01062)
Supplement: Supplementary file 1 [file ao5c01062_si_001.pdf]

Correlating Structural Disorder and Pr^{3+} Emission Dynamics in $Lu_3Al_{2.5-x}Sc_xGa_{2.5}O_{12}$ Crystals: A Comprehensive Structure-Property Investigation

Karol Bartosiewicz[a)*], Wioletta Dewo[b)], Vitali Nagirnyi[c)], Tomasz Runka[b)], Marco Kirm[c)], Takahiko Horiai[d)], Damian Szymanski[e)], Akihiro Yamaji[d,f)], Shunsuke Kurosawa[d),f),g)], Paweł Socha[h)], Jan Pejchal[a)], Vladimir Babin[a)], Robert Kral[a)], Aleksei Kotlov[i)], Akira Yoshikawa[d),f),g)], Martin Nikl[a)]

[a)]Institute of Physics, Academy of Sciences of the Czech Republic, Na Slovance 1999/2, 18200, Praha, Czechia;
[b)]Faculty of Materials Engineering and Technical Physics, Poznan University of Technology, Piotrowo 3, 60965, Poznań, Poland;
[c)]Institute of Physics, University of Tartu, W. Ostwald Str. 1, 50411, Tartu, Estonia;
[d)]New Industry Creation Hatchery Center, Tohoku University, 2-1-1 Katahira Aoba-ku, 9808577, Sendai, Miyagi, Japan;
[e)]Institute of Low Temperature and Structure Research, Polish Academy of Sciences, Okolna 2, 50422, Wrocław, Poland;
[f)]Institute for Materials Research, Tohoku University, 2-1-1 Katahira Aoba-ku, Sendai, 9808577, Sendai, Miyagi, Japan;
[g)]Institute of Laser Engineering, Osaka University, 2-6 Yamadaoka, 5650871, Suita, Osaka, Japan;
[h)]Łukasiewicz Research Network - Institute of Microelectronics and Photonics, Aleja Lotników 32/46, 02-668, Warsaw, Poland;
[i)]Deutsches Elektronen-Synchrotron DESY, Notkestr. 85, Hamburg 22607, Germany

**E-mail of the corresponding author: bartosiewicz@fzu.cz (Karol Bartosiewicz)*

Supporting Information

1. Crystal phase and morphology

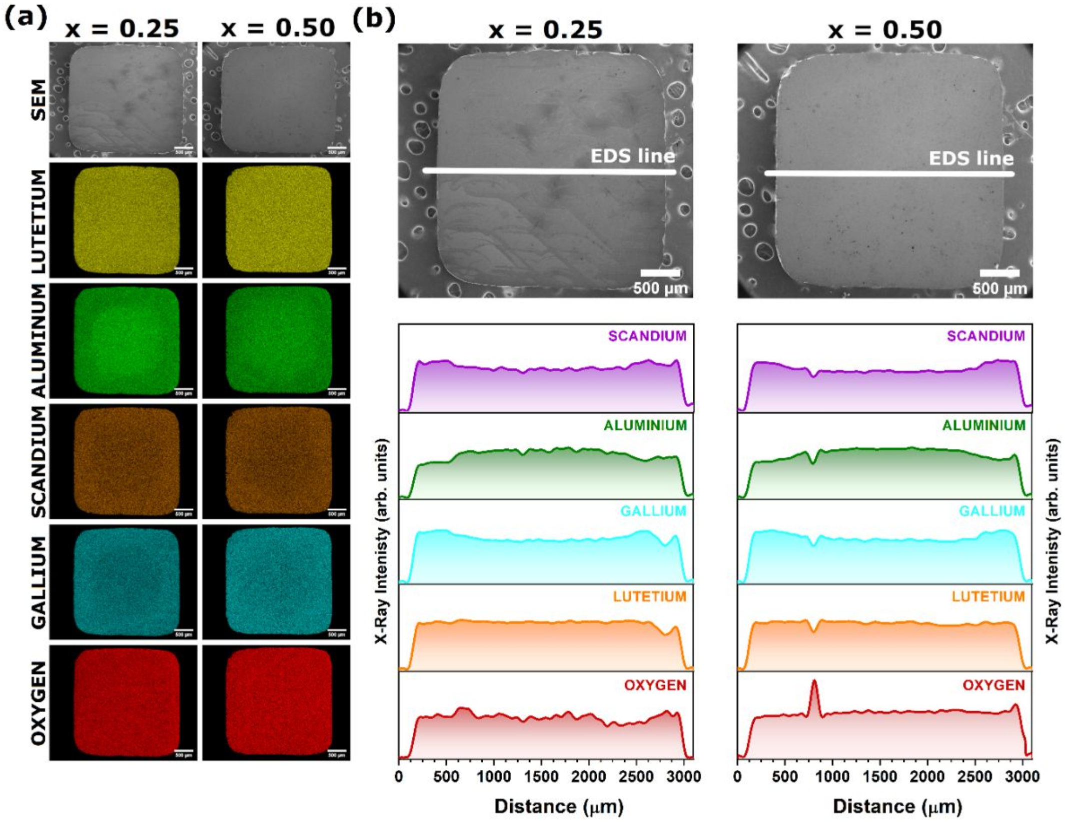

Figure S1. (a) Multielemental EDS mapping of lutetium (Lu, yellow), aluminum (Al, green), scandium (Sc, orange), gallium (Ga, cyan) and oxygen (O, red) of Pr^{3+}-doped $Lu_3Al_{2.5-x}Sc_xGa_{2.5}O_{12}$ crystals, where x = 0.25 and 1.00. (b) Multielemental EDS line profile of the radial distribution of aluminum (Al, green), gallium (Ga, cyan), lutetium (Lu, yellow) and oxygen (O, red) of Pr^{3+}-doped $Lu_3Al_{2.5-x}Sc_xGa_{2.5}O_{12}$ crystals, where x = 0.25 and 0.50.

Figure S1a and Figure S1b illustrate the changes in the radial distribution of Lu, Al, Sc, Ga, and O atoms as a function of increasing Sc^{3+} concentration. The substitution of Sc^{3+} ions induces a systematic improvement in the radial distribution profiles, reflecting enhanced uniformity and structural reorganization within the host lattice. This redistribution of atomic positions significantly influences the material's properties by altering the local coordination environment and crystal field symmetry. These structural modifications impact physical and luminescence characteristics of the material, particularly by promoting increased lattice disorder, modifying energy transfer mechanisms, and ultimately influencing scintillation performance

2. Raman spectroscopy characteristics

Table S1. The experimental wavenumbers (cm^{-1}) and assignment of Raman modes for Pr^{3+}-doped $Lu_3Al_{2.5-x}Sc_xGa_{2.5}O_{12}$ (where x = 0,0 0.5, 1.0). The literature data for LuAG and LuGG single crystals are also included [1-7].

Experimental wavenumber [cm^{-1}]			Literature								
$Lu_3Al_{2.5-x}Sc_xGa_{2.5}O_{12}$:Pr			$Lu_3Al_5O_{12}$				$Lu_3Ga_5O_{12}$			Symmetry	Assignment
x = 0.0	x = 0.5	x = 1.0	8	9	3	10	11	12	13		
118	115	114	107	107	107	107	108	107	106	F_{2g} / E_g	translations RE^{3+} + translations + librations + υ_3 (AlO_4, GaO_4)
130	131	133	130	130	130	130	-	-	-	E_g	
159	162	163	156	156	156	156	166	165	164	F_{2g}	
181	178	174	-	-	-	-	180	180	178	F_{2g}	
240	240	-	240	240	240	240	239	238	236	F_{2g}	
278	276	274	261	261	261	261	269	269	268	F_{2g}	
320	320	320	313	313	313	313	-	-	-	E_g	
333	333	333	330	330	330	330	-	-	-	E_g	
368	360	360	-	-	-	-	356	356	356/359	A_{1g} /E_g	
374	370	367	375	375	375	375	-	-	-	A_{1g}/ F_{2g}	
380	372	372	-	-	-	-	-	-	-	A_{1g}/ F_{2g}	
392	388	386	396	396	396	396	-	394	398	E_g/ F_{2g}	
410	411	410	415	415	417	415	-	419	-	F_{2g}	
555	549	548	551	551	552	551	538	536	533	A_{1g}/ F_{2g}	υ_2 + υ_1 (AlO_4, GaO_4)
-	-	-	-	-	605	-	612	611	616	F_{2g}	
622	620	616	-	-	-	-	625	624	-	F_{2g}	
638	627	624	-	-	-	-	-	-	647	F_{2g} /E_g	
678	666	-	-	-	-	-	-	-	-	-	
786	776	773	773	773	773	773	766	765	765	E_g/A_{1g}/ F_{2g}	υ_4 (AlO_4, GaO_4)
-	-	-	-	777	-	-	-	-	-	E_g/A_{1g}	
836	823	821	-	-	-	-	-	-	-	?	
886	873	869	869	869	869	869	-	-	-	F_{2g}	

3. Absorption and photoluminescence properties

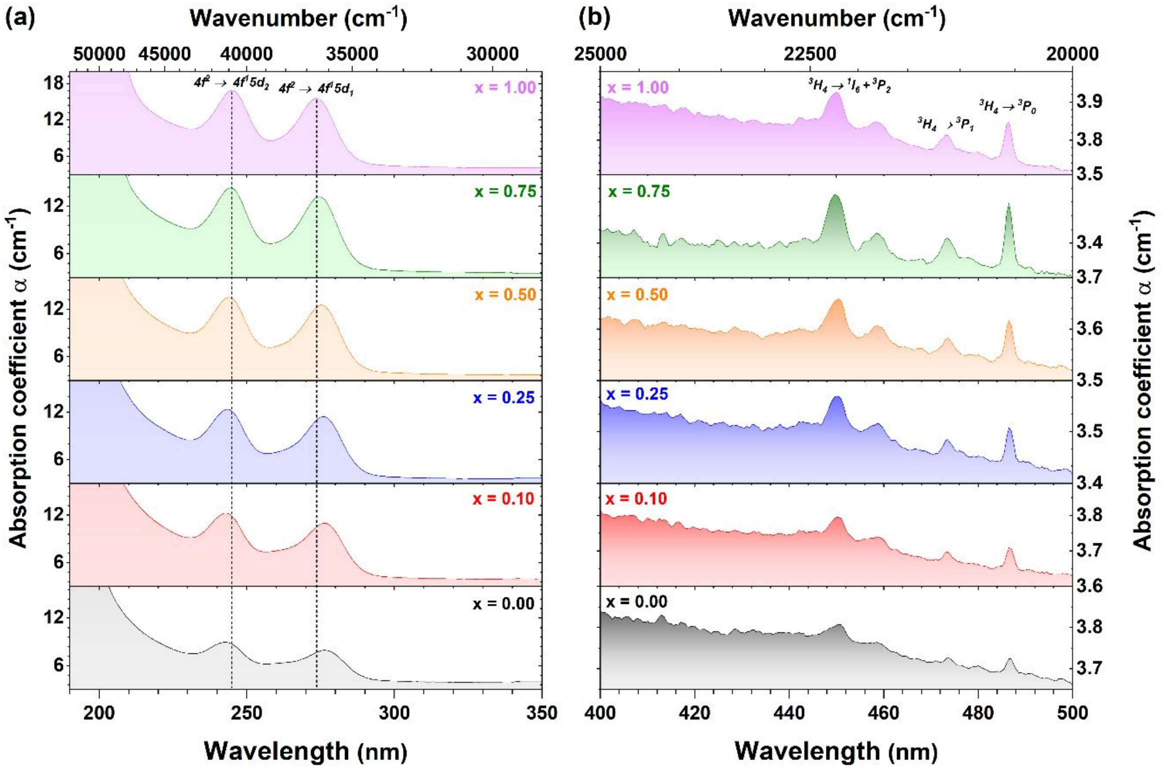

Figure S2. (a,b) Absorption spectra of Pr^{3+}-doped $Lu_3Al_{2.5-x}Sc_xGa_{2.5}O_{12}$ crystals with increasing Sc^{3+} ions concentration. The spectra were measured for the central part of the crystals.

Figure S2 shows the absorption spectra of Pr^{3+}-doped $Lu_3(Al,Sc,Ga)_5O_{12}$ crystals with increasing Sc^{3+} ions concentration. The absorption spectra were recorded for the central part of the crystals. Two characteristic broad absorption bands located at 245 and 275 nm are assigned to the $4f^2 \rightarrow 4f^15d_2$ and $4f^2 \rightarrow 4f^15d_1$ interconfigurational transitions, respectively. The incorporation of large Sc atoms in the crystal structure of $Lu_3Al_{2.5}Ga_{2.5}O_{12}$ causes the shift of the $4f^2 \rightarrow 4f^15d_2$ band towards lower energies (i.e. red shift), while the $4f^2 \rightarrow 4f^15d_1$ band moves to higher energies (blue shift). Furthermore, the fundamental absorption edge shifts toward lower energies with increasing Sc^{3+} ions concentration in the crystal structure of examined samples. This is related to the shrinkage of the band gap and the decrease in crystal field strength imposed by Sc admixing [14]. The spectroscopic analysis reveals also an enhanced

absorption intensity of the $4f^2 \rightarrow 4f^2$ intraconfigurational transitions of Pr^{3+} ions in the Sc-admixed crystals. This enhancement can be attributed to the reduction in local site symmetry surrounding the Pr^{3+} ions, induced by structural perturbations resulting from Sc^{3+} ions incorporation into the host lattice, thereby increasing the transition probability of the above-mentioned transitions. This result is consistent with the multielemental EDS analysis.

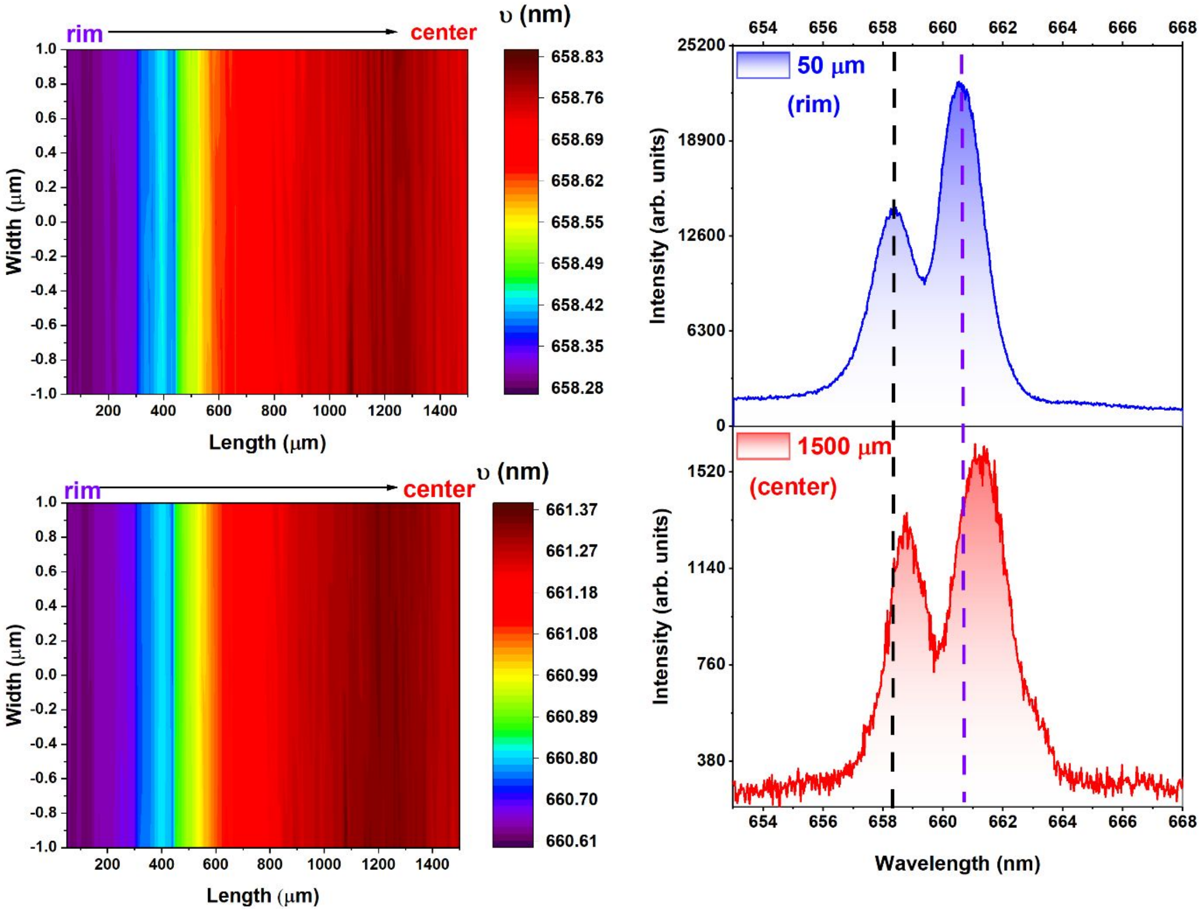

Figure S3. Photoluminescence peak position maps spanning an area from the rim (50 μm) to the center (1500 μm) of the crystal, with a width of 2 μm recorded for the 658 and 660 nm emission bands of the Pr^{3+}-doped $Lu_3Al_{2.4}Sc_{0.1}Ga_{2.5}O_{12}$ crystal (left panels), and the luminescence spectrum of the bands recorded from the rim (50 μm) and the center (1500 μm) of the crystal (right panels).

Figure S3 shows the photoluminescence maximum position maps spanning an area from the rim (50 μm) to the center (1500 μm) of the crystal, with a width of 2 μm recorded for the 658 and 660 nm emission bands. Photoluminescence spectra, which were used for mapping, were recorded from the rim to the core of the crystal. The maps were created for a crystal surface with dimensions of 2 × 1500 μm with a 10 μm step. Two emission lines with maxima at about

658 and 660 nm have been assigned to the $^3P_0 \rightarrow {}^3F_4$ transitions of Pr^{3+} ions were selected in the construction of the maps.

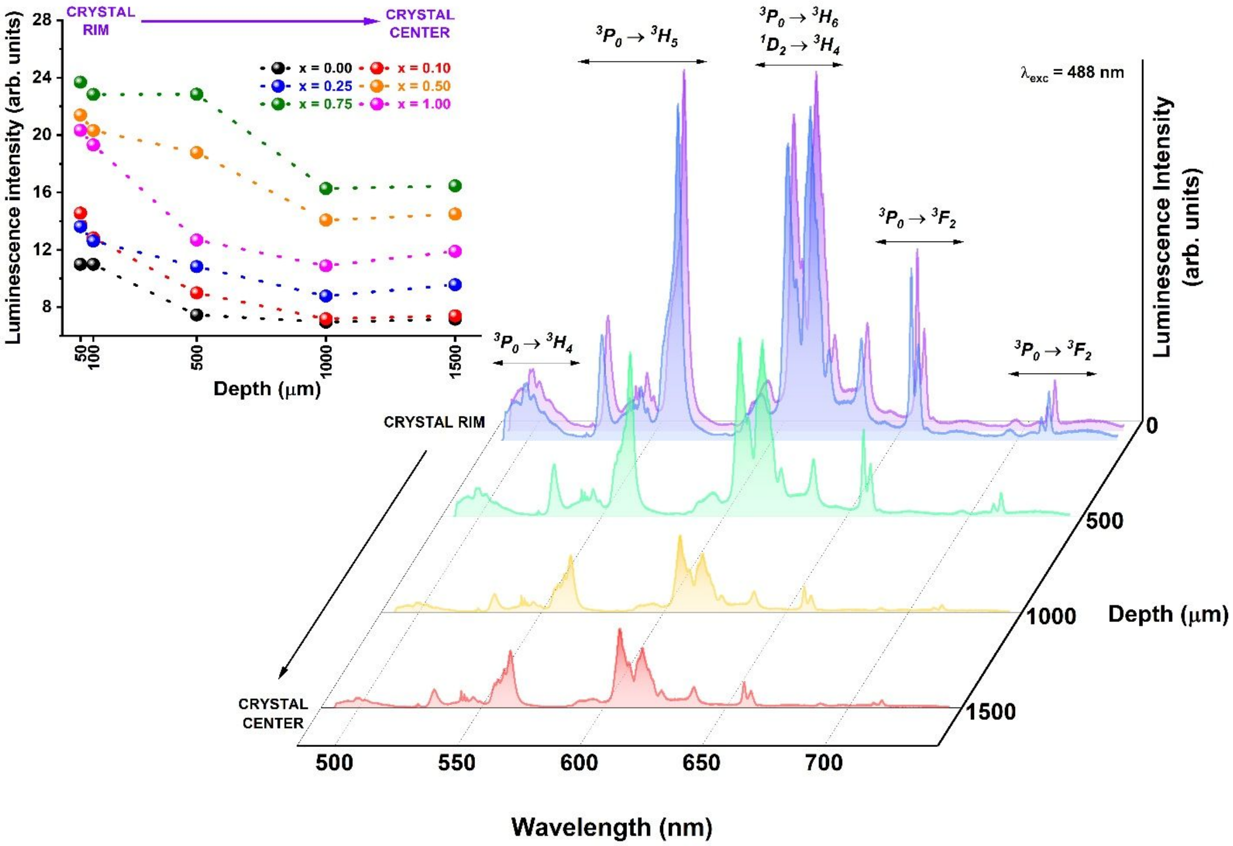

Figure S4. Evolution of the high spatial-resolution luminescence spectra of Pr^{3+}-doped $Lu_3Al_{2.4}Sc_{0.1}Ga_{2.5}O_{12}$ crystal in the cross-section of the sample plate under the excitation at 488 nm line (300 K). The 0 μm distance corresponds to the rim of the crystal cross-section. The left-site inset shows the radial variation in emission intensity corresponding to $^3P_0 \rightarrow {}^3H_6$ and $^1D_2 \rightarrow {}^3H_4$ transitions of Pr^{3+} ions as a function of increasing Sc^{3+} ion concentration.

Figure S4 shows the high spatial resolution luminescence spectra of Pr^{3+}-doped $Lu_3Al_{2.4}Sc_{0.1}Ga_{2.5}O_{12}$ crystal (where x = 0.10) recorded in the visible spectral range (490 – 740 nm) excited at the 488 nm line corresponding to the $^3H_4 \rightarrow {}^3P_0$ transition of Pr^{3+} ions [15]. The spectra were recorded by scanning from the rim to the core of the crystal. The assignment of the observed bands has been made based on the analysis of absorption spectra (see Figure S2 in SI). It can be seen that the intensity of emission bands assigned to the $4f^2 \rightarrow 4f^2$ intraconfigurational transitions of Pr^{3+} ions decreases strongly while moving from the rim of the crystal to its core. This observation confirms that the crystal rim is enriched in Pr^{3+} ions,

while the core is depleted, indicating that the crystal rim has a more perturbed structure than the crystal core. This inhomogeneity in the distribution of Pr^{3+} ions is the result of a low segregation coefficient of Pr atoms in aluminum garnets [16] and the segregation of larger Sc and Ga atoms in the crystal rim. The segregation of Pr^{3+} ions towards the crystal rim is driven by the relaxation of the strain energy generated around dopant cations with sizes larger than the host cation. Furthermore, the energy of the solution and the resistance of the crystal lattice to the incorporation of mismatched elements should also be considered. The differential ionic radii Lu^{3+} ions (Lu^{3+}_{VIII} = 0.977 Å) and large Pr^{3+} ions (Pr^{3+}_{VIII} = 1.126 Å) [17] in eight-fold coordination significantly influence their spatial distribution within the crystal lattice. The larger Pr^{3+} ions exhibit a pronounced affinity for the occupation of the dodecahedral sites within the LuO_8 sublattice. This preferential site occupancy is consistent with the size-dependent cation ordering typically observed in disordered oxide structures. The incorporation of Pr^{3+} ions into the crystal lattice introduces lattice strain due to the mismatch in ionic radii. This strain energy can be relaxed by the segregation of Pr atoms to regions of pre-existing lattice defects, such as the crystal rim, which is inherently more disordered due to surface defects and thermal etching [18]. In addition, the Marangoni melt flow, which occurs at the outer surface of the melt in the molten zone, can amplify this effect. The inset of Figure S4 shows the influence of increasing Sc^{3+} ions concentration on the radial distribution of Pr^{3+} ions. To estimate the radial distribution of Pr^{3+} ions in the Pr^{3+}-doped $Lu_3Al_{2.5-x}Sc_xGa_{2.5}O_{12}$ crystal with different Sc^{3+} ions concentrations, the luminescence intensity of the $^3P_0 \rightarrow {}^3H_6$ and $^1D_2 \rightarrow {}^3H_4$ transitions of Pr^{3+} ions was recorded between the crystal rim and the core for the Pr^{3+}-doped $Lu_3Al_{2.5-x}Sc_xGa_{2.5}O_{12}$ crystal, where x = 0.00 – 1.00. As shown in the inset of Figure S4, the intensity of the emission bands recorded in the spectral region of the $^3P_0 \rightarrow {}^3H_6$ and $^1D_2 \rightarrow {}^3H_4$ transition depends on both Pr^{3+} ions concentration and the disorder degree of the host lattice. At lower concentrations of Sc^{3+} ions x = 0.00 and x = 0.10, luminescence intensity is significantly higher near the rim, suggesting an accumulation of Pr^{3+} ions at the crystal outer regions. The steep decrease in Pr^{3+} luminescence intensity towards the crystal core illustrates that the Pr^{3+} ions are preferentially localized around the rim and exhibit limited diffusion toward the center. This behavior likely arises due to a weak lattice modification and lower facilitation of dopant diffusion in low-Sc environments. As a result, inhomogeneous radial doping is prominent at lower Sc^{3+} ions concentrations. At higher Sc element concentrations x = 0.50, x = 0.75 and x = 1.00, the luminescence distribution exhibits improved uniformity, with a notably smaller radial gradient. This effect reflects enhanced dopant diffusion throughout the crystal, likely due to increased lattice disorder induced by the substitution of Sc^{3+} ions. The lattice disorder

diminishes the energy barriers for the migration of Pr^{3+}, allowing deeper penetration into the crystal. Consequently, the Pr^{3+} ions achieve a more even distribution in the radial profile. To better understand the changes in the radial distribution of the Al, Sc, and Ga atoms, photoluminescence maps were measured for the Pr^{3+}-doped $Lu_3Al_{2.4}Sc_{0.1}Ga_{2.5}O_{12}$ crystal refer to Figure S3 in SI. The photoluminescence mapping shows that the positions of both lines shift towards longer wavelengths while moving from the rim to the core of the crystal. The most significant changes in line positions occur up to 600 μm from the crystal rim, while at approaching the crystal core, the changes become much smaller. This phenomenon is related to the change in the concentration of Pr^{3+} ions as well as in the radial fluctuation of Al, Sc, and Ga elements influencing the local environment of Pr^{3+} ions. This result is consistent with EDS elemental mapping and EDS line profiles presented in Figure 2 and Figure 3, respectively, where the highest gradient in the concentration of Al, Sc, and Ga atoms is observed up to 600 μm from the crystal rim.

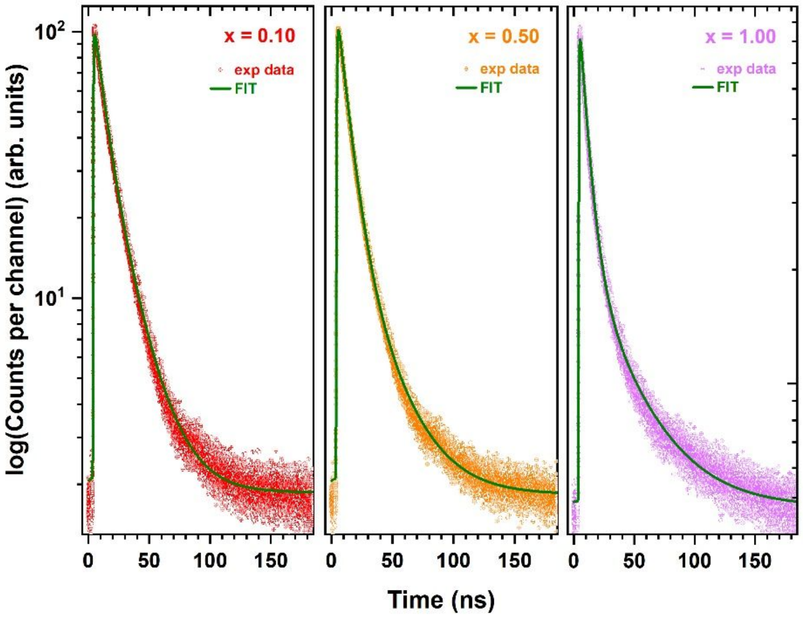

Figure S5. Photoluminescence decay curves for the $4f^15d_1^1 \rightarrow 4f^2$ interconfigurational transition of the Pr^{3+} ions (λ_{exc} = 244 nm, λ_{emi} = 299 nm) in the Pr^{3+}-doped $Lu_3Al_{2.5-x}Sc_xGa_{2.5}O_{12}$ crystals where x = 0.10, 0.50 and 1.00 recorded at the P66 beamline.

Table S2. Experimental decay times of the $5d_1^1$ emitting level of Pr^{3+} ions in the Pr^{3+}-doped $Lu_3Al_{2.5-x}Sc_xGa_{2.5}O_{12}$ crystals with increasing Sc^{3+} ions concentration. FIT stands for the

fraction of the total intensity (FTI) contribution of each component to the overall decay time value.

**Sc^{3+} ions concentration in the crystal** (chemical formula units)	**Decay time values**			
	**τ_1 (ns)**	**FTI of τ_1 (%)**	**τ_2 (ns)**	**FTI of τ_2 (%)**
0.00	-	-	18.0	100(*)
0.10	8.3	57	20.1	43
0.25	5.3	37	13.8	63
0.50	5.3	47	13.6	53
0.75	5.1	72	14.3	28
1.00	5.1	76	14.1	24

(*) The decay time for the Pr^{3+}-doped $Lu_3Al_{2.5}Ga_{2.5}O_{12}$ crystal (where x = 0.00) was measured using an Edinburgh Instruments FLS920 spectrofluorometer with an H_2 nanosecond discharge lamp as an excitation source. The abbreviation FTI represents the fraction of the total intensity.

Figure S5 presents the decay kinetics of Pr^{3+} luminescence, recorded under 244 nm excitation at P66. The decay constants (decay times of separate components and the fraction of their initial intensities in percentage) obtained by a two-exponential fit of the experimental data for all samples are summarized in Table S2. The Pr^{3+}-doped $Lu_3Al_{2.5}Ga_{2.5}O_{12}$ crystal (where x = 0.00) was excited with a pulsed H_2 lamp and monitored emission at 310 nm. The decay curves were fitted with single or double exponential functions according to the following equation:

$$I(t) = \sum I_i \exp\left(\frac{-t}{\tau_i}\right) + B, \qquad \text{(ES1)}$$

where I is luminescence intensity, I_i is the intensity of i component at 0 ns, τ_i is its decay time, t is time and B is the background intensity. As can be seen in Figure S5, the decay curve for the Pr^{3+}-doped $Lu_3Al_{2.5}Ga_{2.5}O_{12}$ crystal (where x = 0.00) shows a single exponential profile with a decay constant of approximately 18 ns, which is slightly shorter than the decay time reported for the Pr^{3+}-doped $Lu_3Al_5O_{12}$ crystal equal to 20 ns [16]. The introduction of Sc^{3+} ions has a significant effect on the photoluminescence decay time of Pr^{3+}-doped $Lu_3Al_{2.5-x}Sc_xGa_{2.5}O_{12}$ crystals. The samples containing Sc exhibit a double-exponential decay profile, characterized by the presence of two distinct decay components. This dual decay behavior suggests that multiple relaxation pathways are involved in the Pr^{3+} $4f^15d_1^1 \rightarrow 4f^2$ emission process. Notably, the first component possesses a decay time similar to that observed in the Sc-free sample (as shown in Table S2). In contrast, the second component exhibits a significantly

faster decay rate. Furthermore, the fraction of the total intensity (FTI) contribution of each component to the overall decay time was estimated from the relation:

$$FTI = \frac{I_i \tau_i}{\sum_i I_i \tau_i} \times 100\% \tag{ES2}$$

This calculation provides the weight fraction of each component, which, in the case of these crystals, shows significant variation. This variation suggests the presence of complex mechanisms contributing to the quenching of the $5d_1^1$ excited state of the Pr^{3+} ions.

Table S3. Comparison of maximum band position and full width at half maximum (FWHM) of the $5d_14f_1 \rightarrow 4f^2$ interconfigurational emission bands of Pr^{3+} ions across various multicomponent garnet host materials.

**Materials**	**Maximum band position (nm)**	**FWHM (nm)**	**Method**	**Year**
$Lu_3Al_5O_{12}$:Pr	310	89	µ-Pulling down	2024[15]
$Y_3Al_5O_{12}$:Pr	320	90	µ-Pulling down	2013[19]
$Lu_{2.25}Y_{0.75}Al_5O_{12}$:Pr	318	93	Czochralski	2019[20]
$Lu_2Y_1Al_3Ga_2O_{12}$:Pr	315	101	µ-Pulling down	2012[21]
$Gd_3Al_3Ga_2O_{12}$:Pr	330	30	µ-Pulling down	2012[22]
$Y_3Al_3Ga_2O_{12}$:Pr	313	100	µ-Pulling down	2013[23]
$Gd_2Y_1Al_3Ga_2O_{12}$:Pr	315	25	µ-Pulling down	2013[23]
$Lu_3Al_{2.5}Ga_{2.5}O_{12}$:Pr	305	96	µ-Pulling down	This work
$Lu_3Al_{2.4}Sc_{0.1}Ga_{2.5}O_{12}$:Pr	304	98	µ-Pulling down	
$Lu_3Al_{2.25}Sc_{0.25}Ga_{2.5}O_{12}$:Pr	303	100	µ-Pulling down	
$Lu_3Al_2Sc_{0.5}Ga_{2.5}O_{12}$:Pr	302	103	µ-Pulling down	
$Lu_3Al_{1.75}Sc_{0.75}Ga_{2.5}O_{12}$:Pr	302	106	µ-Pulling down	
$Lu_3Al_{1.5}Sc_1Ga_{2.5}O_{12}$:Pr	300	109	µ-Pulling down	

Table S3 summarizes the spectroscopic parameters, specifically the maximum band position and full width at half maximum, of the $5d_14f^1 \rightarrow 4f^2$ interconfigurational emission transitions of Pr^{3+} ions in diverse multicomponent garnet host matrices. The data demonstrates that Sc substitution exhibits the most pronounced effect on increasing structural disorder within the host lattice. This finding has significant implications for the rational design of disordered host lattice matrices, particularly in the development of advanced optical materials. The enhanced

structural disorder can be strategically exploit to optimize absorption coefficients and broaden emission bands, crucial parameters for improving the performance of laser materials and phosphors. These modifications could lead to substantial improvements in quantum efficiency and overall device performance in various photonic applications, including solid-state lighting, displays, and laser systems.

4. Scintillation properties

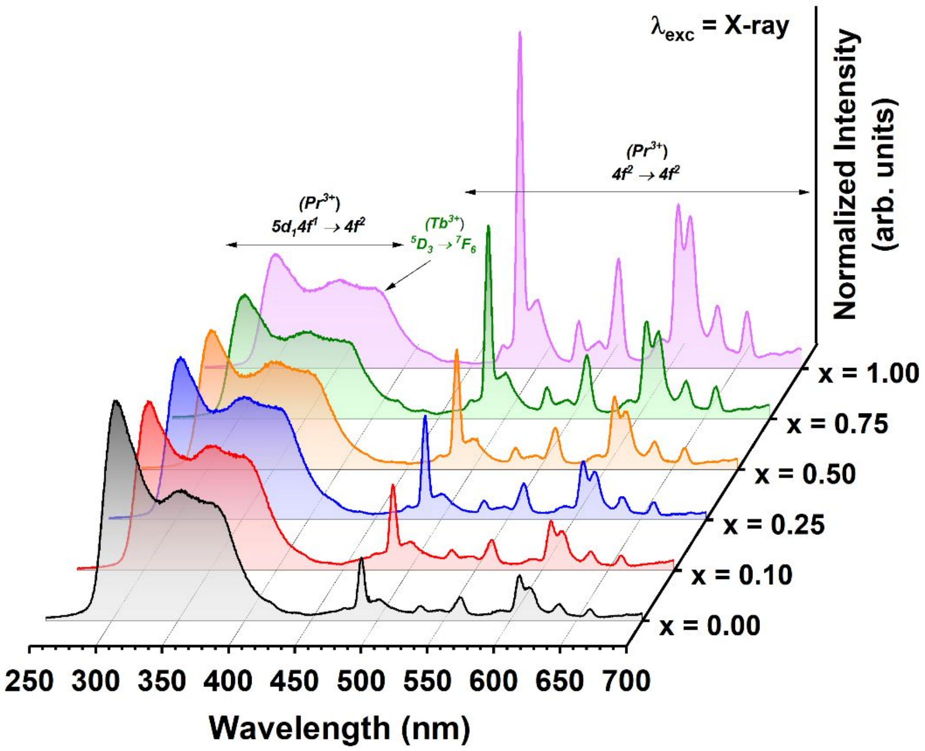

Figure S6. Radioluminescence spectra of Pr^{3+}-doped $Lu_3Al_{2.5-x}Sc_xGa_{2.5}O_{12}$ single crystals with increasing Sc^{3+} ions concentration.

The radioluminescence spectra of the Pr^{3+}-doped $Lu_3(Al,Sc,Ga)_5O_{12}$ crystals are shown in Figure S6. Broad band recorded in the 280 – 420 nm spectral range belongs to the $4f^15d_1 \rightarrow 4f^2$ interconfigurational transition of Pr^{3+} ions. The $4f^2 \rightarrow 4f^2$ intraconfigurational transitions of Pr^{3+} ions are observed in the visible spectra region between 480 and 750 nm. Similar to the case of direct interconfigurational excitation (4f→$5d_1$ excitation of Pr^{3+} ions, see Figure 6,

under X-ray excitation, the intensity of $5d_1 4f^1 \rightarrow 4f^2$ interconfigurational transitions decreases, while the $4f^2 \rightarrow 4f^2$ intraconfigurational transitions rises with the increasing Sc^{3+} ions concentration. Additionally, in the 383 – 450 nm spectral range were also recorded emission lines which were assigned to the Tb^{3+} trace impurity.

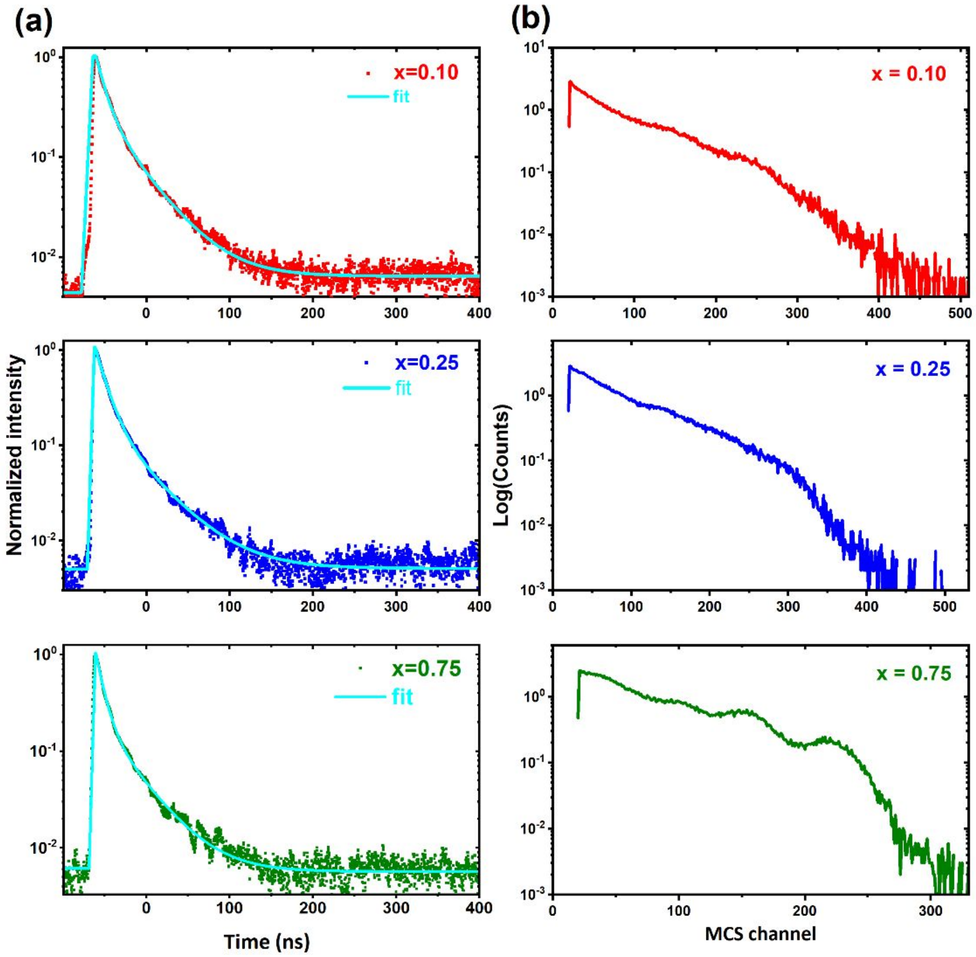

Figure S7. (a) Scintillation decay curves and (b) pulse-height spectra of Pr^{3+}-doped $Lu_3Al_{2.5-x}Sc_xGa_{2.5}O_{12}$ crystals with increasing Sc^{3+} ions concentration under γ-rays excitation from the ^{137}Cs radioisotope.

Figure S7a shows the scintillation decay curves of Pr^{3+} emission in $Lu_3(Al,Sc,Ga)_5O_{12}$ crystals. The decay constants were determined by the convolution of the instrumental response with a selected function, which was double-exponential for all crystals. The fast component (τ_1) of the Pr^{3+}-doped $Lu_3Al_{2.5}Ga_{2.5}O_{12}$ crystal derived from the fit is equal to 22 ns, which is a typical decay constant for the Pr^{3+}-doped LuAG single crystal [16]. The slower component (τ_2) equal to70 ns is due to a delayed radiative recombination process [2]. The fast and slow decay constants,

in Sc-containing crystals, both accelerate continuously with the increasing Sc^{3+} ions concentrations. These observations align with the photoluminescence findings, lending credence to the proposed mechanisms underlying the quenching of fast luminescence previously elucidated. The pulse-height spectra presented in Figure S7b demonstrate a reduction in light yield (LY) values, indicating that a fraction of the ionized electrons undergo delayed radiative recombination with Pr^{4+} centers after their return from the conduction band (CB). This temporal phenomenon extends beyond the established integration time window for LY measurements, resulting in delayed recombination luminescence that is not captured within the standard detection parameters. These observations provide compelling evidence for the presence of electron trapping-detrapping processes that influence the scintillation kinetics of the crystals.

5. Thermally stimulated luminescence

According to the single-trap model [24], the traps found in thermoluminescence above 250 K are considered deep traps that can reduce the scintillation yield at 300 K. The rapid sequential capture of charge carriers followed by their radiative recombination at Pr^{3+} ions is a potential mechanism for host-to-ion energy transfer in the $Lu_3Al_5O_{12}$ host. In addition, there is a delayed, trap-mediated route for energy transfer. Since Pr^{3+} ions are likely to capture valence band holes efficiently [25], it can be assumed that the traps responsible for the TL signals in Figure S8 are related to the electron traps. The TL glow curve measurements, conducted following X-ray irradiation at 77 K, provide comprehensive insights into the trap state distribution and their crucial role in the scintillation kinetics. The TL glow curves show very similar profiles, with different intensities and slightly different band positions, which do not show a linear dependence on the change of Sc^{3+} ions concentration. The observed irregularities in TL glow curve profiles can be attributed to radial compositional heterogeneity in the distribution of the Al, Sc, and Ga atoms within the crystal structure [26-28]. The TL glow curves in the 77 – 200 K temperature range in all examined crystals can be related to the electron traps associated with the antisite Lu^{x}_{Al} traps [28]. This observation is supported by relatively low TL intensity and is consistent with the previous report [28]. The origin of TL glow peaks in the 200 – 300 K range, particularly in aluminum garnets, is highly complex. Extensive literature indicates that these TL peaks may arise from several mechanisms: (i) tunneling-driven processes[7, 29-31], (ii) a distribution of trapping states[32-36] and (iii) the presence of trace impurities such as Yb, Cr, or Mn[35, 37-39]. Given the complexity of these interacting factors, a comprehensive and precise

elucidation of the origin of the traps responsible for the TL glow peak in this temperature range would require extensive investigation, which is beyond the scope of the present study. The dominant TL bands in the 280 – 500 K temperature range and the peak with the maximum at around 350 K are probably related to the oxygen vacancies ($V_O^{\bullet\bullet}$) [26, 40]. Quantitative analysis of the integrated TL intensities within the temperature range of 80 – 490 K (Figure S8b) reveals that the magnitude of trap contributions exhibits no direct correlation with the substantial decrease in scintillation light yield, as demonstrated in Figure 8b and Table 2. Instead, the observed reduction in LY values demonstrates a strong correlation with the significant acceleration of photoluminescence decay times, which is proportional to increasing Sc^{3+} ion concentration, as illustrated in Figure S5 and Table S2. This finding suggests that the primary mechanism affecting the scintillation efficiency is more closely associated with the modification of excited state dynamics rather than trap-related phenomena. Hence, the TL data provide an important conclusion: the incorporation of Sc^{3+} ions exerts a minimal effect on the formation of stable shallow and deep trapping centers. This suggests that the perturbation of the local potential, induced by the substitution of Sc^{3+} ions into the dodecahedral and octahedral sites of the crystal lattice, creates localized trapping centers where electrons and holes become bound, leading to the formation of an excitonic state. Notably, this excitonic state is highly unstable, promoting radiative transitions that facilitate the transfer of excitation energy to Pr^{3+} ions. This mechanism is consistent with photoluminescence spectra observed under synchrotron radiation excitation, which reveal energy transfer processes associated with Sc incorporation. The findings highlight the interplay between Sc^{3+}-induced lattice perturbations and exciton-mediated energy transfer processes in the host matrix. The presence of Sc has a negligible effect on the formation of stable shallow and deep trapping centers, an observation of significant importance for the design of novel oxide scintillators and dosimetric materials.

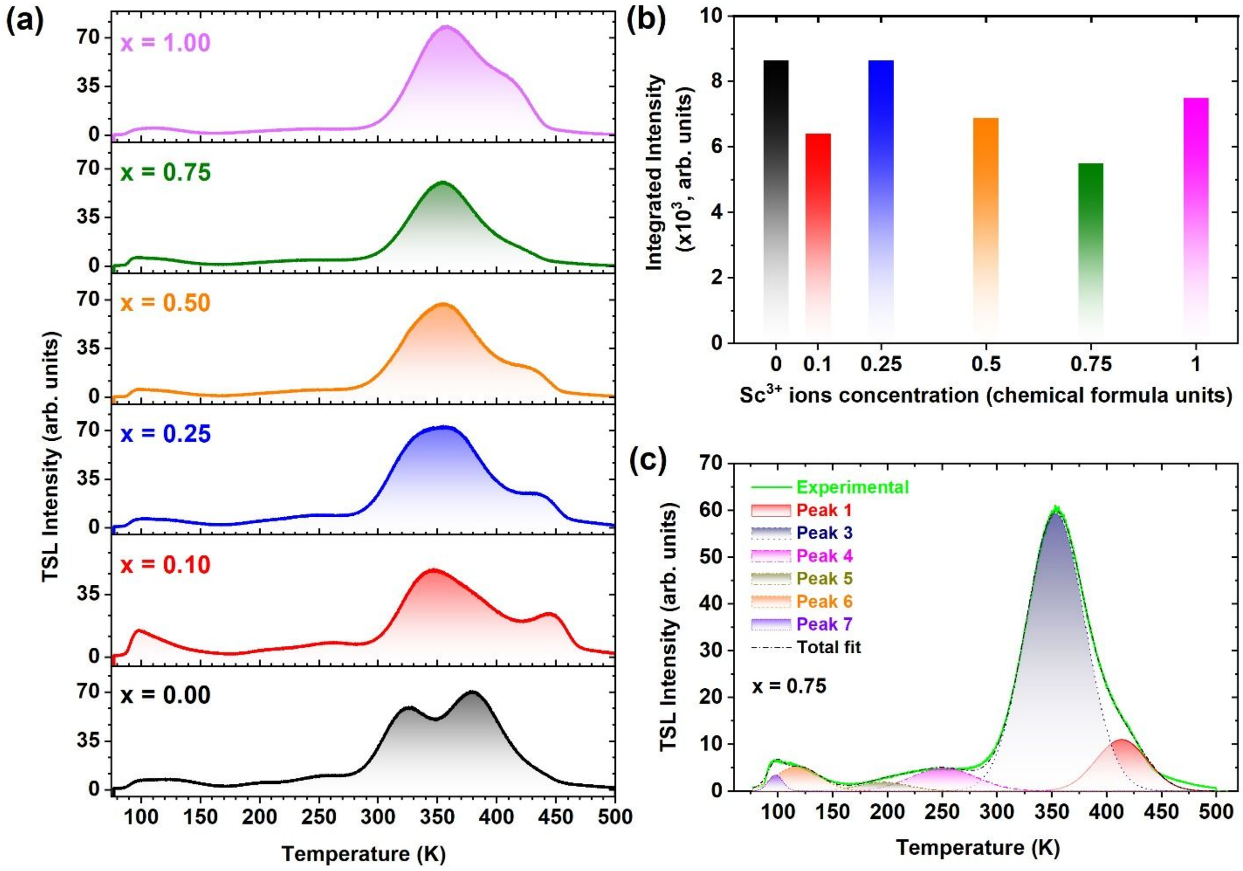

Figure S8. (a) Thermoluminescence glow curve of Pr^{3+}-doped $Lu_3Al_{2.5-x}Sc_xGa_{2.5}O_{12}$ crystals with increasing Sc^{3+} ions concentration after X-ray irradiation at 77 K. (b) Integrated intensity of TL glow curbed between 80 and 490 K. (c) Thermoluminescence glow curve of Pr^{3+}-doped $Lu_3Al_{1.75-x}Sc_{0.75}Ga_{2.5}O_{12}$ crystal deconvoluted into single peaks.

A comprehensive analysis of the TL glow curves was conducted through their deconvolution into single peaks. This process was carried out using the GlowFit software [41] which applies peak fitting based on the first-order kinetic model. The deconvolution approach allows for the separation of overlapping glow peaks, providing detailed insights into the trapping and recombination processes underlying the TL response. To achieve a satisfactory fit with a figure of merit ranging from 3% to 6%, up to six-seven components with distinct energy levels were required. The exception was the sample without Sc, which required seven distinct energy levels. The deconvoluted into single peaks revealed that, upon Sc incorporation, two maxima centered at 380 K and 322 K overlapped and converged into a single maximum around 375 – 353 K in the Sc admixed crystals. The trap parameters are summarized in Table S4 and S5. The trap depths range from 1.63 eV (deep traps) to 0.22 eV (shallow traps) and are comparable

across all samples. The determined frequency factors lie predominantly within the range of 10^7 to 10^{11} s^{-1}, consistent with first-order thermoluminescent kinetics.

In addition, the Urbach methos[42, 43] assumes that the maximum temperature of the TL glow peak corresponds to a temperature slightly below that at which the probability of electron escape from a trap reaches unity. Urbach derived a simple expression for the trap activation energy E, given by

$$E = 23kT_{max} \quad \text{(ES3)}$$

Where E is the trap activation energy (eV), k = 8.617×10^{-5} eV/K is the Boltzmann constant, and T_{max} is the temperature (in Kelvin) at which the TL intensity is maximized. It should be noted that the numerical coefficient is dependent on the frequency factor s, and therefore the calculated E values are approximate, serving only as first-order estimates. Table S5 summarizes the activation energies determined at the glow peak maxima. Furthermore, the activation energies calculated for individual glow peaks differ significantly from those obtained through deconvolution into single first-order kinetic peaks (see Table S4). This discrepancy arises because the Urbach expression uses a fixed frequency factor ($s = 2.9 \times 10^9$ s^{-1}), despite s being temperature-dependent. Consequently, the deconvolution into a single first-order kinetic peaks model yields a more precise estimation of the activation energies than the Urbach approach.

Table S4. The trap parameters detected in Pr^{3+}-doped $Lu_3Al_{-x}Sc_xGa_{2.5}O_{12}$ crystals with increasing Sc^{3+} ions concentration obtained from deconvolution into single peaks include the peak temperature (T_{max}), activation energy (E), and frequency factor (s).

**Sc^{3+} ions concentration** (chemical formula units)	**Parameters**	**TL peak number**						
		**1**	**2**	**3**	**4**	**5**	**6**	**7**
	T_{max} (K)	432	380	322	257	195	125	100
0.00	E (eV)	1.57	1.12	0.99	0.79	0.53	0.37	0.25
	s (s^{-1})	7.11 x 10^{11}	1.00 x 10^{11}	0.61 x 10^{11}	9.01 x 10^{11}	3.41 x 10^{7}	4.64 x 10^{7}	2.83 x 10^{7}
	T_{max} (K)	429	none	353	256	210	119	99
0.10	E (eV)	1.56	none	0.89	0.68	0.57	0.32	0.24
	s (s^{-1})	1.76 x 10^{11}	none	2.41 x 10^{11}	6.24 x 10^{11}	3.27 x 10^{7}	1.17 x 10^{7}	2.39 x 10^{7}
	T_{max} (K)	436	none	351	247	209	123	99
0.25	E (eV)	1.59	none	0.68	0.59	0.54	0.37	0.22
	s (s^{-1})	1.55 x 10^{11}	none	4.34 x 10^{11}	6.95 x 10^{11}	9.49 x 10^{7}	5.64 x 10^{7}	1.14 x 10^{7}
	T_{max} (K)	440	none	354	248	210	119	99
0.50	E (eV)	1.63	none	0.66	0.59	0.53	0.34	0.24
	s (s^{-1})	3.24 x 10^{11}	none	1.17 x 10^{11}	1.21 x 10^{11}	1.01 x 10^{7}	2.17 x 10^{7}	7.93 x 10^{7}
	T_{max} (K)	417	none	354	247	198	118	98
0.75	E (eV)	1.43	none	0.67	0.57	0.54	0.32	0.25
	s (s^{-1})	8.58 x 10^{11}	none	4.82 x 10^{11}	1.90 x 10^{11}	2.95 x 10^{7}	1.45 x 10^{7}	1.03 x 10^{7}
1.00	T_{max} (K)	411	none	357	251	196	121	101
	E (eV)	1.39	none	0.70	0.59	0.52	0.35	0.28
	s (s^{-1})	3.17 x 10^{11}	none	1.39 x 10^{11}	8.10 x 10^{11}	5.59 x 10^{7}	0.45 x 10^{7}	2.84 x 10^{7}

Table S5. The trap parameters detected in Pr^{3+}-doped $Lu_3Al_{-x}Sc_xGa_{2.5}O_{12}$ crystals with increasing Sc^{3+} ions concentration obtained from Urbach method include the peak temperature (T_{max}) and activation energy (E).

**Sc^{3+} ions concentration** (chemical formula units)	**Parameters**	**TL peak number**						
		**1**	**2**	**3**	**4**	**5**	**6**	**7**
0.00	T_{max} (K)	432	380	322	257	195	125	100
	E (eV)	0.86	0.75	0.64	0.51	0.39	0.25	0.20
0.10	T_{max} (K)	429	none	353	256	210	119	99
	E (eV)	0.85	none	0.70	0.51	0.42	0.24	0.20
0.25	T_{max} (K)	436	none	351	247	209	123	99
	E (eV)	0.86	none	0.70	0.49	0.41	0.24	0.20
0.50	T_{max} (K)	440	none	354	248	210	119	99
	E (eV)	0.87	none	0.70	0.49	0.42	0.24	0.20
0.75	T_{max} (K)	417	none	354	247	198	118	98
	E (eV)	0.83	none	0.70	0.49	0.39	0.23	0.19
1.00	T_{max} (K)	411	none	357	251	196	121	101
	E (eV)	0.81	none	0.71	0.50	0.39	0.24	0.20

Acknowledgments

This project has received funding from the European Union's Horizon Europe research and innovation programme under the Marie Skłodowska-Curie Actions COFUND, Physics for Future, grant agreement No 101081515, the National Science Centre Poland (NCN) No.: 2020/39/D/ST3/02711, Estonian Research Council grants PRG629, PRG2733 and RVTT3 as well as the GIMRT program of the Institute for Materials Research, Tohoku University Proposal No. 202306-RDKYA-0533. The Estonian Ministry of Education and Research (TK210), and Research Project of the Polish Ministry of Education and Science 0511/SBAD/2351 and Czech Science Foundation project GA24-14580L are also acknowledged with thanks. We acknowledge DESY (Hamburg, Germany), a member of the Helmholtz Association HGF, for the provision of experimental facilities. Parts of this research were carried out at the PETRA III storage ring. Beamtime was allocated to the proposal I-20211463 EC.

References

(1) Zapadlík, O.; Pejchal, J.; Kučerková, R.; Beitlerová, A.; Nikl, M. Composition-Engineered GSAG Garnet: Single-Crystal Host for Fast Scintillators. *Crystal Growth & Design* **November 16, 2021**, *21* (12). DOI: 10.1021/acs.cgd.1c01007.

(2) Nikl, M.; Yoshikawa, A.; Kamada, K.; Nejezchleb, K.; Stanek, C. R.; Mares, J. A.; Blazek, K. Development of LuAG-based scintillator crystals – A review. *Progress in Crystal Growth and Characterization of Materials* **2013**, *59* (2), 47-72. DOI: 10.1016/j.pcrysgrow.2013.02.001.

(3) Papagelis, K.; Ves, S. Infrared spectroscopy and lattice dynamical calculations of $Gd_3Al_5O_{12}$, $Tb_3Al_5O_{12}$ and $Lu_3Al_5O_{12}$ single crystals. *J Phys Chem Solids* **2003**, *64* (4), 599-605. DOI: Pii S0022-3697(02)00359-1

Doi 10.1016/S0022-3697(02)00359-1.

(4) Kasamatsu, T.; Sekita, H.; Kuwano, Y.; Kasamatsu, T.; Sekita, H.; Kuwano, Y. Temperature dependence and optimization of 970-nm diode-pumped Yb:YAG and Yb:LuAG lasers. *Applied Optics, Vol. 38, Issue 24, pp. 5149-5153* **1999-08-20**, *38* (24). DOI: 10.1364/AO.38.005149.

(5) Hart, D. W.; Jani, M.; Barnes, N. P. Room-temperature lasing of end-pumped Ho:$Lu_3Al_5O_{12}$. *Opt Lett* **1996**, *21* (10), 728-730. DOI: 10.1364/ol.21.000728 From NLM PubMed-not-MEDLINE.

(6) Petrosyan, A. G.; Ovanesyan, K. L.; Shirinyan, G. O.; Sargsyan, R. V.; Dujardin, C.; Pedrini, C. Site occupation and solubility limit of Sc in $Lu_3Al_5O_{12}$. *J Cryst Growth* **2012**, *338* (1), 143-146. DOI: 10.1016/j.jcrysgro.2011.11.034.

(7) Nikl, M.; Vedda, A.; Fasoli, M.; Fontana, I.; Laguta, V. V.; Mihokova, E.; Pejchal, J.; Rosa, J.; Nejezchleb, K. Shallow traps and radiative recombination processes in $Lu_3Al_5O_{12}$:Ce single crystal scintillator. *Physical Review B* **2007-11-21**, *76* (19). DOI: 10.1103/PhysRevB.76.195121.

(8) Papagelis, K.; Arvanitidis, J.; Kanellis, G.; Kourouklis, G. A.; Ves, S. High Pressure Raman Study of Lu3Al5O12. *physica status solidi (b)* **1999/01/01**, *211* (1). DOI: 10.1002/(SICI)1521-3951(199901)211:1<301::AID-PSSB301>3.0.CO;2-R.

(9) Papagelis, K.; Arvanitidis, J.; Kanellis, G.; Ves, S.; Kourouklis, G. A.; Papagelis, K.; Arvanitidis, J.; Kanellis, G.; Ves, S.; Kourouklis, G. A. High-pressure effects on the Raman spectrum andthe force constants of the rare-earth aluminium garnets(RE3Al5O12). *Journal of Physics: Condensed Matter* **2002-04-04**, *14* (15). DOI: 10.1088/0953-8984/14/15/303.

(10) Chiriu, D.; Ricci, P. C.; Carbonaro, C. M.; Anedda, A.; Aburish-Hmidat, M.; Grosu, A.; Lorrai, P. G.; Fortin, E. Vibrational properties of mixed $(Y_3Al_5O_{12})_x$ - $(Y_3Sc_2Ga_3O_{12})_{1-x}$ crystals. *Journal of Applied Physics* **2006**, *100* (3). DOI: 10.1063/1.2220473

10.1063/1.2220473.
(11) Venkatramu, V.; Giarola, M.; Mariotto, G.; Enzo, S.; Polizzi, S.; Jayasankar, C. K.; Piccinelli, F.; Bettinelli, M.; Speghini, A. Nanocrystalline lanthanide-doped $Lu_3Ga_5O_{12}$ garnets: interesting materials for light-emitting devices. *Nanotechnology* **2010**, *21* (17), 175703. DOI: 10.1088/0957-4484/21/17/175703 From NLM PubMed-not-MEDLINE.
(12) Monteseguro, V.; Rodriguez-Hernandez, P.; Ortiz, H. M.; Venkatramu, V.; Manjon, F. J.; Jayasankar, C. K.; Lavin, V.; Munoz, A. Structural, elastic and vibrational properties of nanocrystalline lutetium gallium garnet under high pressure. *Phys Chem Chem Phys* **2015**, *17* (14), 9454-9464. DOI: 10.1039/c4cp05903d From NLM PubMed-not-MEDLINE.
(13) Song, J. J.; Klein, P. B.; Wadsack, R. L.; Selders, M.; Mroczkow.S; Chang, R. K. Raman-Active Phonons in Aluminum, Gallium, and Iron Garnets. *J Opt Soc Am* **1973**, *63* (9), 1135-1140. DOI: Doi 10.1364/Josa.63.001135.
(14) Ding, S.; Ding, S.; Ren, H.; Ren, H.; Zou, Y.; Zou, Y.; Liu, W.; Liu, W.; Zhang, Q.; Zhang, Q. Single crystal growth and property investigation of Dy3+ and Tb3+ co-doped Gd3Sc2Al3O12 (GSAG): multiple applications for GaN blue LD pumped all-solid-state yellow lasers and UV or blue light chip excited solid-state lighting. *Journal of Materials Chemistry C* **2021/08/05**, *9* (30). DOI: 10.1039/D1TC02294F.
(15) Bartosiewicz, K.; Albini, B.; Szymański, D.; Socha, P.; Horiai, T.; Yoshino, M.; Yamaji, A.; Kurosawa, S.; Kucerkova, R.; Galinetto, P.; et al. Engineering atomic size mismatch in Pr^{3+}, La^{3+} codoped $Lu_3Al_5O_{12}$ garnet single crystals for tailored structure and functional properties. *J Alloy Compd* **2024**, *985*. DOI: 10.1016/j.jallcom.2024.174078.
(16) Ogino, H.; Yoshikawa, A.; Nikl, M.; Kamada, K.; Fukuda, T. Scintillation characteristics of Pr-doped $Lu_3Al_5O_{12}$ single crystals. *J Cryst Growth* **2006**, *292* (2), 239-242. DOI: 10.1016/j.jcrysgro.2006.04.021.
(17) Shannon, R. D. Revised effective ionic radii and systematic studies of interatomic distances in halides and chalcogenides. *Acta Crystallographica Section A* **1976**, *32* (5), 751-767. DOI: 10.1107/s0567739476001551.
(18) Bartosiewicz, K.; Takahiko, H.; Akihiro, Y.; Yoshikawa, A.; Kurosawa, S.; Yoshino, M.; Zorenko, Y. Effects of La doping on the crystal growth, phase stability and scintillation properties of Lu3Al5O12 single crystals. *Materials Science And Engineering: B* **2020**, *261*, 114677-114677. DOI: 10.1016/j.mseb.2020.114677.
(19) Sreebunpeng, K.; Chewpraditkul, W.; Babin, V.; Nikl, M.; Nejezchleb, K. Scintillation response of $Y_3Al_5O_{12}:Pr^{3+}$ single crystal scintillators. *Radiation Measurements* **2013**, *56*, 94-97. DOI: 10.1016/j.radmeas.2013.04.013.
(20) Foster, C.; Wu, Y.; Stand, L.; Koschan, M.; Melcher, C. L. Effect of lithium codopant concentration on the luminescence properties of $(Lu_{0.75}Y_{0.25})_3Al_5O_{12}:Pr^{3+}$ single crystals: Before and after air annealing. *J Lumin* **2019**, *216*, 116751. DOI: 10.1016/j.jlumin.2019.116751 (acccessed 2021).
(21) Kamada, K.; Yanagida, T.; Pejchal, J.; Nikl, M.; Endo, T.; Kousuke, T.; Fujimoto, Y.; Akihiro, F.; Yoshikawa, A. Improvement of Scintillation Properties in Pr Doped $Lu_3Al_5O_{12}$ Scintillator by Ga and Y Substitutions. *IEEE Transactions on Nuclear Science* **2012**, *59* (5), 2130-2134. DOI: 10.1109/tns.2012.2191621 (acccessed 2024).
(22) Kamada, K.; Yanagida, T.; Pejchal, J.; Nikl, M.; Endo, T.; Tsutumi, K.; Usuki, Y.; Fujimoto, Y.; Fukabori, A.; Yoshikawa, A. Growth and scintillation properties of Pr doped $Gd_3(Ga,Al)_5O_{12}$ single crystals. *J Cryst Growth* **2012**, *352* (1), 84-87. DOI: 10.1016/j.jcrysgro.2012.02.002.
(23) Nikl, M.; Kamada, K.; Kurosawa, S.; Yokota, Y.; Yoshikawa, A.; Pejchal, J.; Babin, V. Luminescence and scintillation mechanism in Ce^{3+} and Pr^{3+} doped $(Lu,Y,Gd)_3(Ga,Al)_5O_{12}$ single crystal scintillators. *Phys Status Solidi C* **2013**, *10* (2), 172-175. DOI: 10.1002/pssc.201200499.
(24) Drozdowski, W.; Dorenbos, P.; Drozdowska, R.; Bos, A. J. J.; Poolton, N. R. J.; Tonelli, M.; Alshourbagy, M. Effect of Electron Traps on Scintillation of Praseodymium Activated Lu-Al-O- | IEEE

Journals & Magazine | IEEE Xplore. *IEEE Transactions on Nuclear Science* **2009**, *56* (1). DOI: 10.1109/TNS.2008.2011269.
(25) Dorenbos, P.; Dorenbos, P. Systematic behaviour in trivalent lanthanide charge transfer energies. *Journal of Physics: Condensed Matter* **2003-11-25**, *15* (49). DOI: 10.1088/0953-8984/15/49/018.
(26) Bartosiewicz, K.; Markovskyi, A.; Horiai, T.; Szymański, D.; Kurosawa, S.; Yamaji, A.; Yoshikawa, A.; Zorenko, Y. A study of Mg2+ ions effect on atoms segregation, defects formation, luminescence and scintillation properties in Ce3+ doped Gd3Al2Ga3O12 single crystals. *Journal of Alloys and Compounds* **2022**, *905*, 164154-164154. DOI: 10.1016/j.jallcom.2022.164154.
(27) Bartosiewicz, K. Elemental Fluctuation in Gd3Al2Ga3O12:Ce Crystals Imposed by Li+ and Mg2+ Co-Doping: The Impact on Defects, Luminescence, and Scintillation Properties. *Metals* **2023**, *13* (2), 422-422. DOI: 10.3390/met13020422 (acccessed 2024).
(28) Nikl, M.; Mihokova, E.; Pejchal, J.; Vedda, A.; Fasoli, M.; Fontana, I.; Laguta, V. V.; Babin, V.; Nejezchleb, K.; Yoshikawa, A.; et al. Scintillator Materials—Achievements, Opportunities, and Puzzles | IEEE Journals & Magazine | IEEE Xplore. *IEEE Transactions on Nuclear Science* **2008**, *55* (3). DOI: 10.1109/TNS.2007.913480.
(29) Mihokova, E.; Babin, V.; Pejchal, J.; Cuba, V.; Barta, J.; Popovich, K.; Schulman, L. S.; Yoshikawa, A.; Nikl, M. Afterglow and Quantum Tunneling in Ce-Doped Lutetium Aluminum Garnet. *IEEE Transactions on Nuclear Science* **2018**, *65* (8), 2085-2089. DOI: 10.1109/tns.2018.2823582.
(30) Mihokova, E.; Schulman, L. S. Low temperature delayed recombination and trap tunneling. *J Phys Condens Matter* **2015**, *27* (7), 075501. DOI: 10.1088/0953-8984/27/7/075501 From NLM PubMed-not-MEDLINE.
(31) Mihóková, E.; Babin, V.; Bartosiewicz, K.; Schulman, L. S.; Václav, Č.; Miroslav, K.; Nikl, M. Low temperature delayed recombination decay in scintillating garnets. *Opt Mater* **2015**, *40*, 127-131. DOI: 10.1016/j.optmat.2014.12.011 (acccessed 2024).
(32) Fasoli, M.; Vedda, A.; Nikl, M.; Jiang, C.; Uberuaga, B. P.; Andersson, D. A.; McClellan, K. J.; Stanek, C. R. Band-gap engineering for removing shallow traps in rare-earth Lu3Al5O12garnet scintillators using Ga3+doping. *Physical Review B* **2011**, *84* (8). DOI: 10.1103/physrevb.84.081102 (acccessed 2021).
(33) Ueda, J.; Ueda, J.; Dorenbos, P.; Dorenbos, P.; Bos, A. J. J.; Bos, A. J. J.; Kuroishi, K.; Kuroishi, K.; Tanabe, S.; Tanabe, S. Control of electron transfer between Ce3+ and Cr3+ in the Y3Al5−xGaxO12 host via conduction band engineering. *Journal of Materials Chemistry C* **2015/05/28**, *3* (22). DOI: 10.1039/C5TC00546A.
(34) Khanin, V.; Venevtsev, I.; Chernenko, K.; Rodnyi, P.; van Swieten, T.; Spoor, S.; Boerekamp, J.; Wieczorek, H.; Vrubel, I.; Meijerink, A.; et al. Variation of the conduction band edge of $(Lu,Gd)_3(Ga,Al)_5O_{12}$:Ce garnets studied by thermally stimulated luminescence. *J Lumin* **2019/07/01**, *211*, 48-53. DOI: 10.1016/j.jlumin.2019.03.013.
(35) Khanin, V. M.; Venevtsev, I.; Chernenko, K.; Tukhvatulina, T.; Rodnyi, P. A.; Spoor, S.; Boerekamp, J.; van Dongen, A.-M.; Buettner, D.; Wieczorek, H.; et al. Influence of 3d Transition Metal Impurities on Garnet Scintillator Afterglow. *Crystal Growth & Design* **2020**, *20* (5), 3007-3017. DOI: 10.1021/acs.cgd.9b01660.
(36) Wieczorek, H.; Khanin, V.; Ronda, C.; Boerekamp, J.; Spoor, S.; Steadman, R.; Venevtsev, I.; Chernenko, K.; Tukhvatulina, T.; Vrubel, I.; et al. Band Gap Variation and Trap Distribution in Transparent Garnet Scintillator Ceramics. *Ieee Transactions on Nuclear Science* **2020**, *67* (8), 1934-1945. DOI: 10.1109/Tns.2020.3001303.
(37) Ueda, J.; Hashimoto, A.; Takemura, S.; Ogasawara, K.; Dorenbos, P.; Tanabe, S. Vacuum referred binding energy of 3d transition metal ions for persistent and photostimulated luminescence phosphors of cerium-doped garnets. *J Lumin* **2017**, *192*, 371-375. DOI: 10.1016/j.jlumin.2017.07.006.
(38) Khanin, V. M.; Rodnyi, P. A.; Wieczorek, H.; Ronda, C. R. Electron traps in $Gd_3Ga_3Al_2O_{12}$:Ce garnets doped with rare-earth ions. *Technical Physics Letters* **2017**, *43* (5), 439-442. DOI: 10.1134/S1063785017050042.

(39) Khanin, V. M.; Vrubel, II; Polozkov, R. G.; Shelykh, I. A.; Venevtsev, I. D.; Meijerink, A.; Wieczorek, H.; Boerekamp, J.; Spoor, S.; Rodnyi, P. A.; et al. Modeling and Assessment of Afterglow Decay Curves from Thermally Stimulated Luminescence of Complex Garnets. *J Phys Chem A* **2019**, *123* (9), 1894-1903. DOI: 10.1021/acs.jpca.8b11778 From NLM PubMed-not-MEDLINE.
(40) Zhong, J. P.; Liang, H. B.; Su, Q.; Zhou, J. Y.; Wang, J. Y. Spectroscopic properties of vacancies and trap levels in $Lu_3Al_5O_{12}:Ce^{3+}$ crystals. *T Nonferr Metal Soc* **2009**, *19* (6), 1628-1633. DOI: 10.1016/S1003-6326(09)60080-7.
(41) Puchalska, M.; Bilski, P. GlowFit—a new tool for thermoluminescence glow-curve deconvolution. *Radiation Measurements* **2006**, *41* (6), 659-664. DOI: 10.1016/j.radmeas.2006.03.008.
(42) Urbach, F. Zur lumineszenz der alkalihalogenide. *Sitzungsberichte Akad. der Wiss. Wien* **1930**, *139* (728), 363-372.
(43) Vasilis Pagonis, G. K., Claudio Furetta. *Numerical and Practical Exercises in Thermoluminescence*; Springer, 2006. DOI: https://doi.org/10.1007/0-387-30090-2.
